# Correction: Predatory journals: Perception, impact and use of Beall’s list by the scientific community–A bibliometric big data study

**DOI:** 10.1371/journal.pone.0296582

**Published:** 2024-01-02

**Authors:** Georg Richtig, Marina Berger, Max Koeller, Markus Richtig, Erika Richtig, Jörg Scheffel, Marcus Maurer, Frank Siebenhaar

There are errors in the Funding section. The correct Funding statement is: Georg Richtig received funding from the Austrian Science Fund FWF (W1241) and the Medical University Graz through the PhD Program Molecular Fundamentals of Inflammation (DK-MOLIN). We acknowledge financial support from the Open Access Publication Fund of Charité –Universitätsmedizin Berlin and the German Research Foundation (DFG).The funders had no role in study design, data collection and analysis, decision to publish, or preparation of the manuscript.
