# Supplementary material for: Predatory journals: Perception, impact and use of Beall’s list by the scientific community–A bibliometric big data study
Source: PLoS One. 2023 Jul 7;18(7):e0287547. doi: 10.1371/journal.pone.0287547 (PMC10328228; doi:10.1371/journal.pone.0287547)
Supplement: S2 File — (PPTX) [file pone.0287547.s002.pptx]

## Slide 1
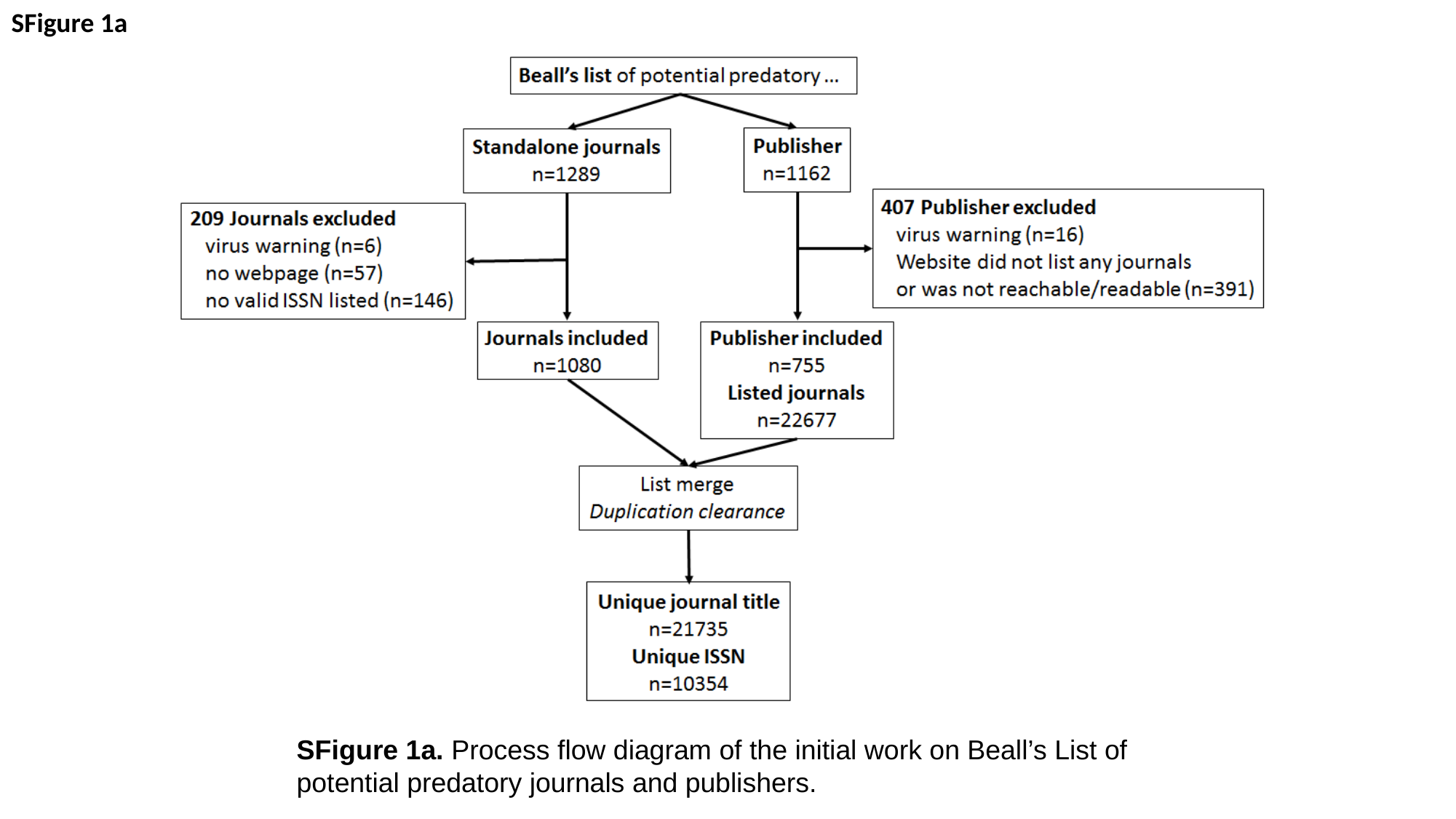

SFigure 1a
SFigure 1a. Process flow diagram of the initial work on Beall’s List of potential predatory journals and publishers.

## Slide 2
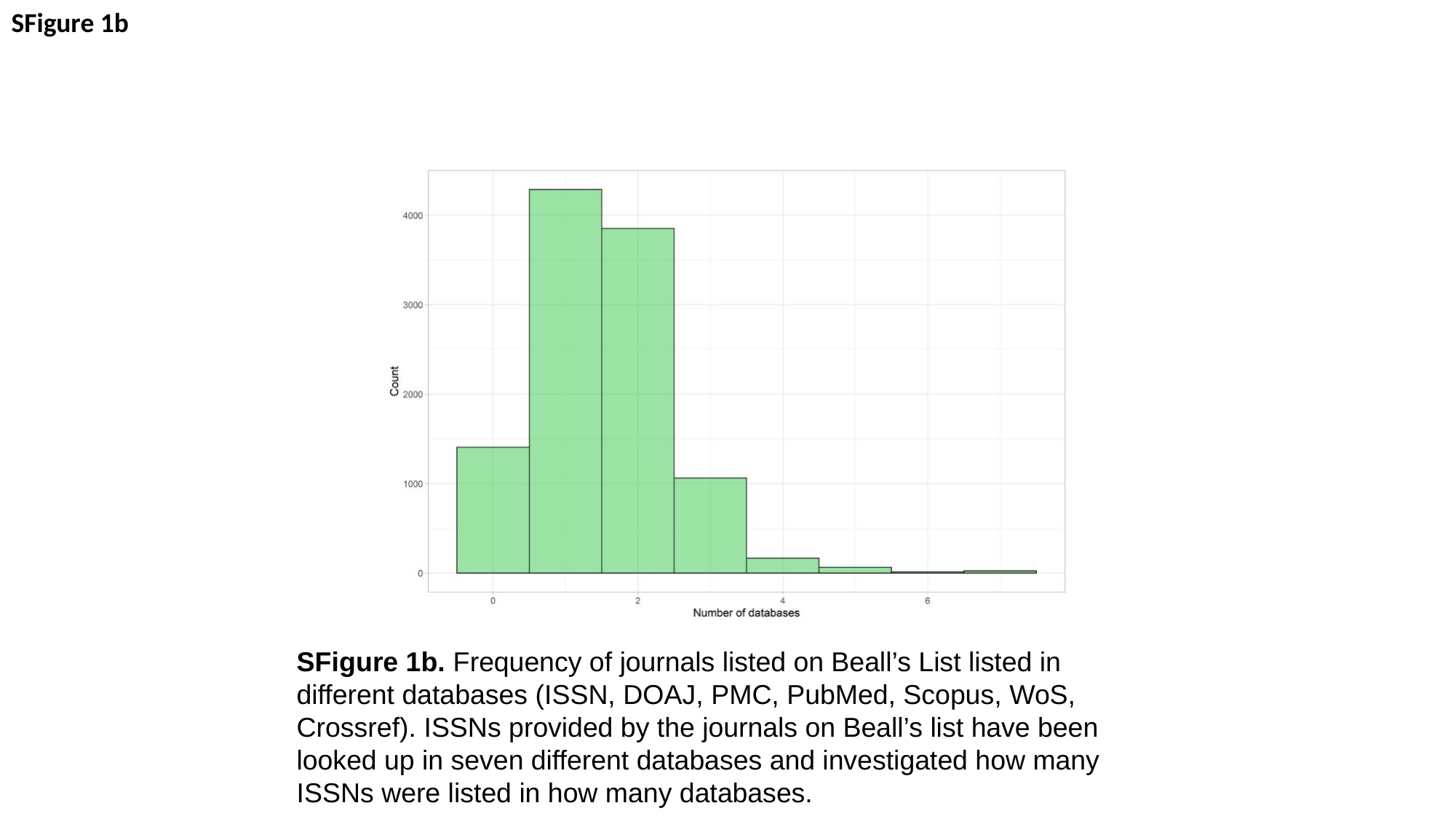

SFigure 1b
SFigure 1b. Frequency of journals listed on Beall’s List listed in different databases (ISSN, DOAJ, PMC, PubMed, Scopus, WoS, Crossref). ISSNs provided by the journals on Beall’s list have been looked up in seven different databases and investigated how many ISSNs were listed in how many databases.

## Slide 3
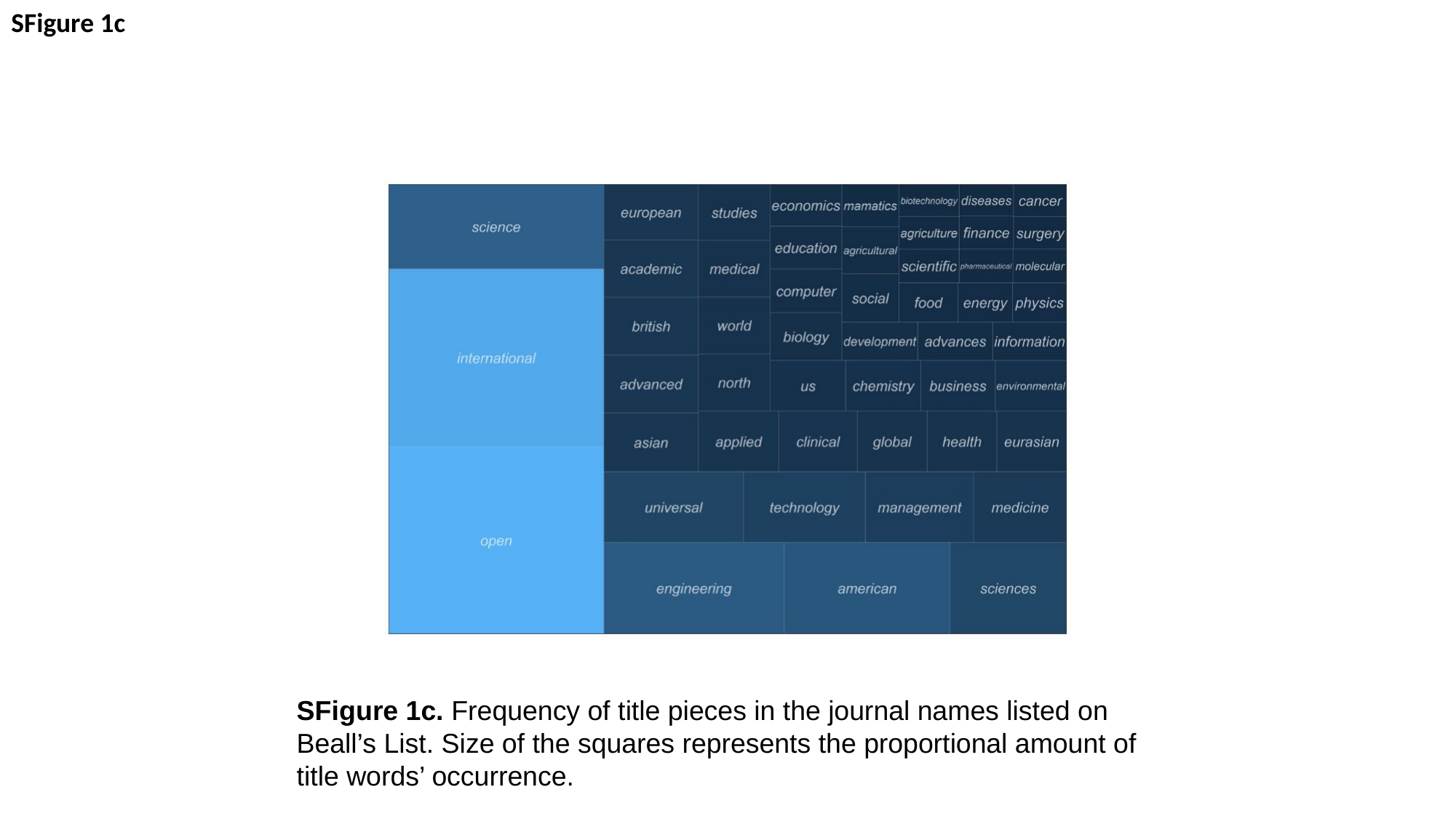

SFigure 1c
SFigure 1c. Frequency of title pieces in the journal names listed on Beall’s List. Size of the squares represents the proportional amount of title words’ occurrence.

## Slide 4
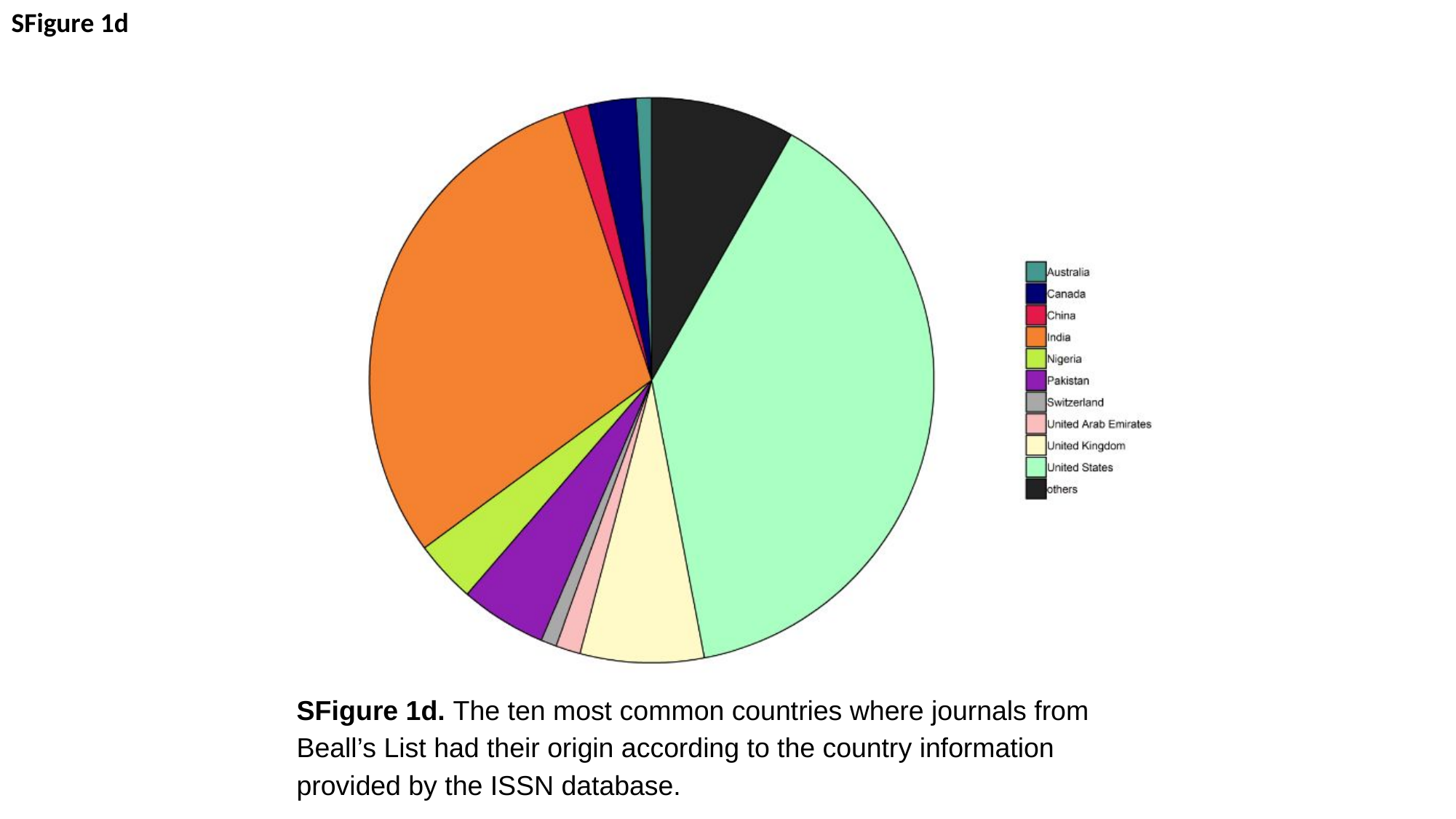

SFigure 1d
SFigure 1d. The ten most common countries where journals from Beall’s List had their origin according to the country information provided by the ISSN database.

## Slide 5
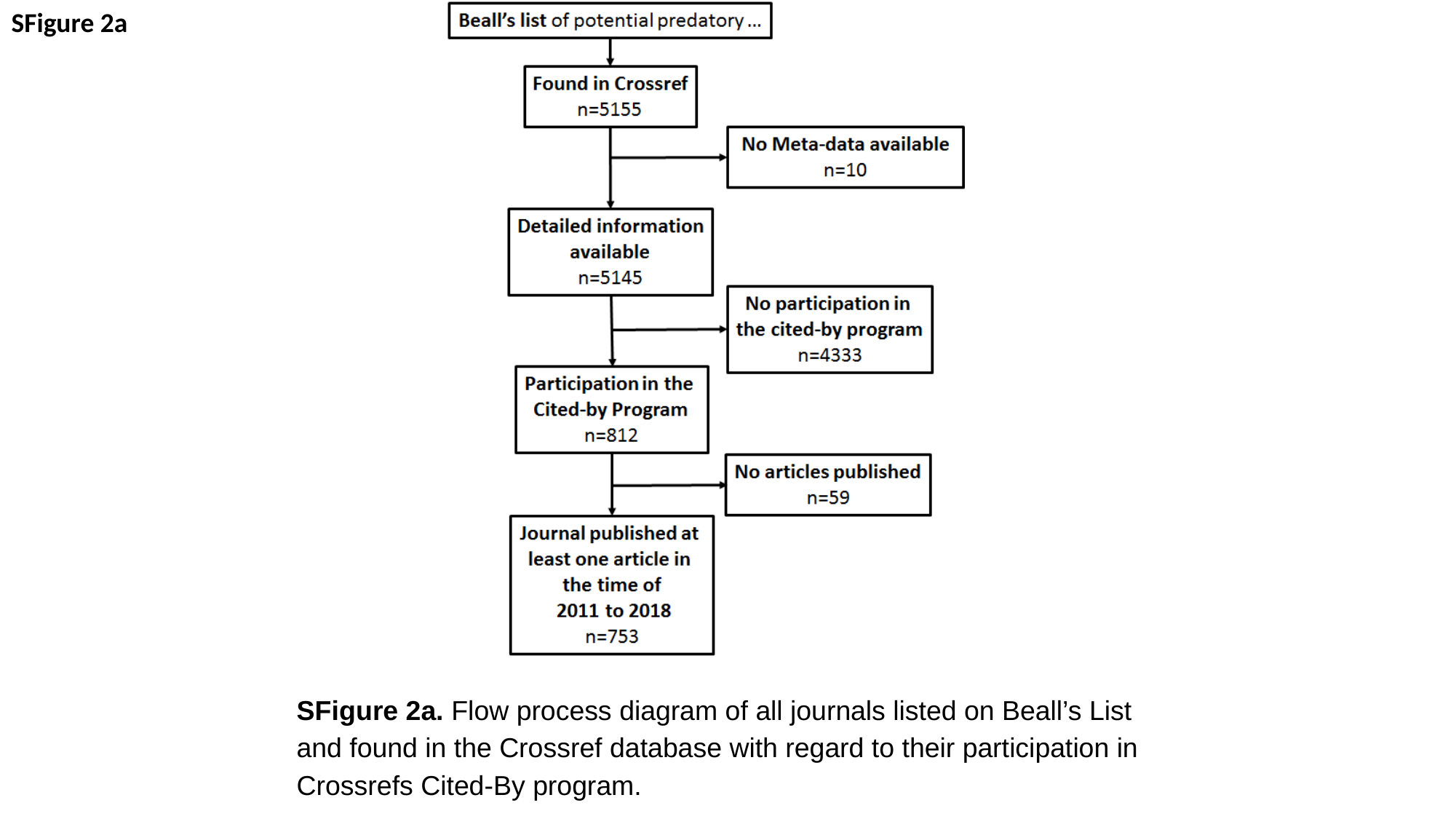

SFigure 2a
SFigure 2a. Flow process diagram of all journals listed on Beall’s List and found in the Crossref database with regard to their participation in Crossrefs Cited-By program.

## Slide 6
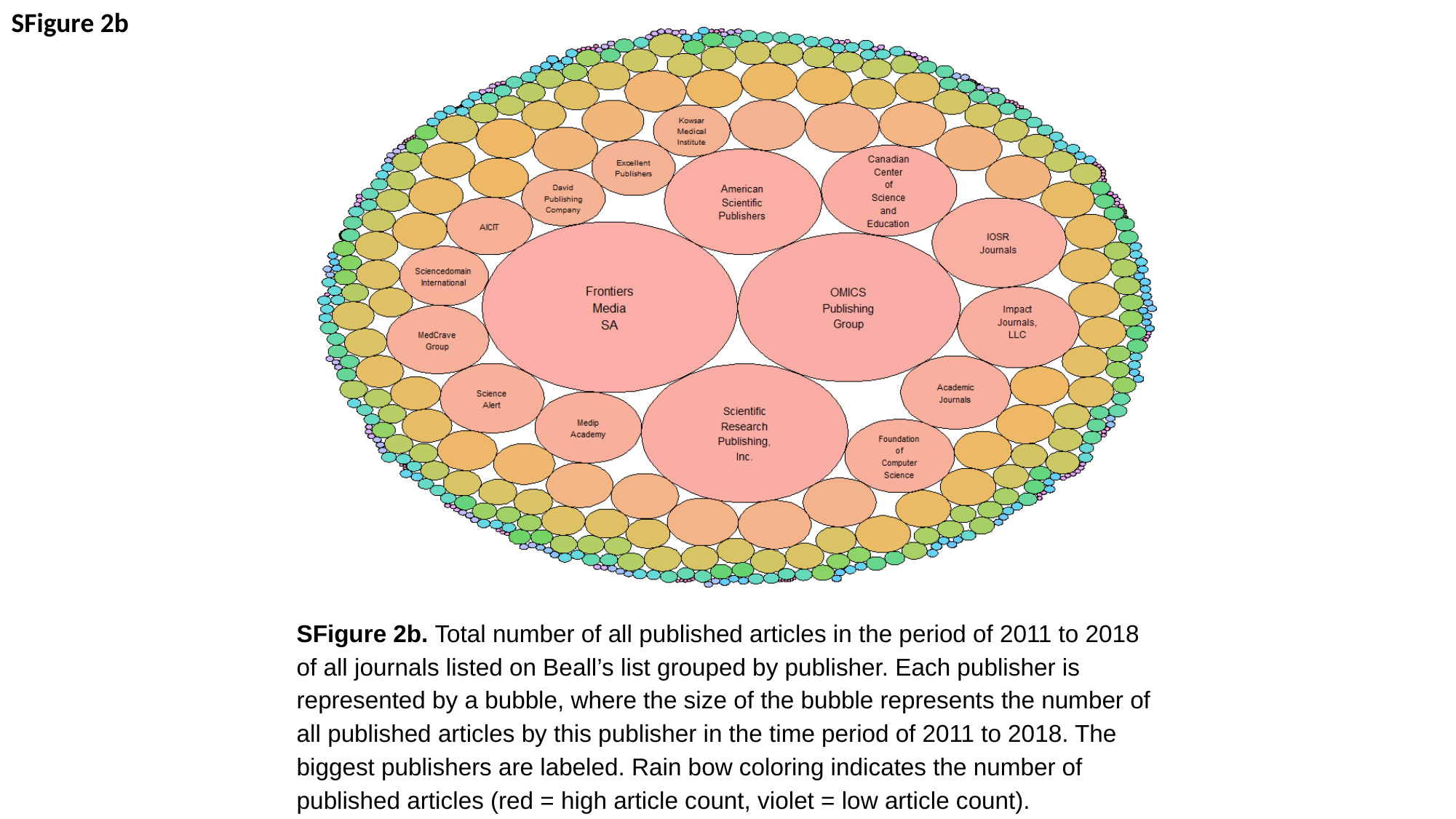

SFigure 2b
SFigure 2b. Total number of all published articles in the period of 2011 to 2018 of all journals listed on Beall’s list grouped by publisher. Each publisher is represented by a bubble, where the size of the bubble represents the number of all published articles by this publisher in the time period of 2011 to 2018. The biggest publishers are labeled. Rain bow coloring indicates the number of published articles (red = high article count, violet = low article count).

## Slide 7
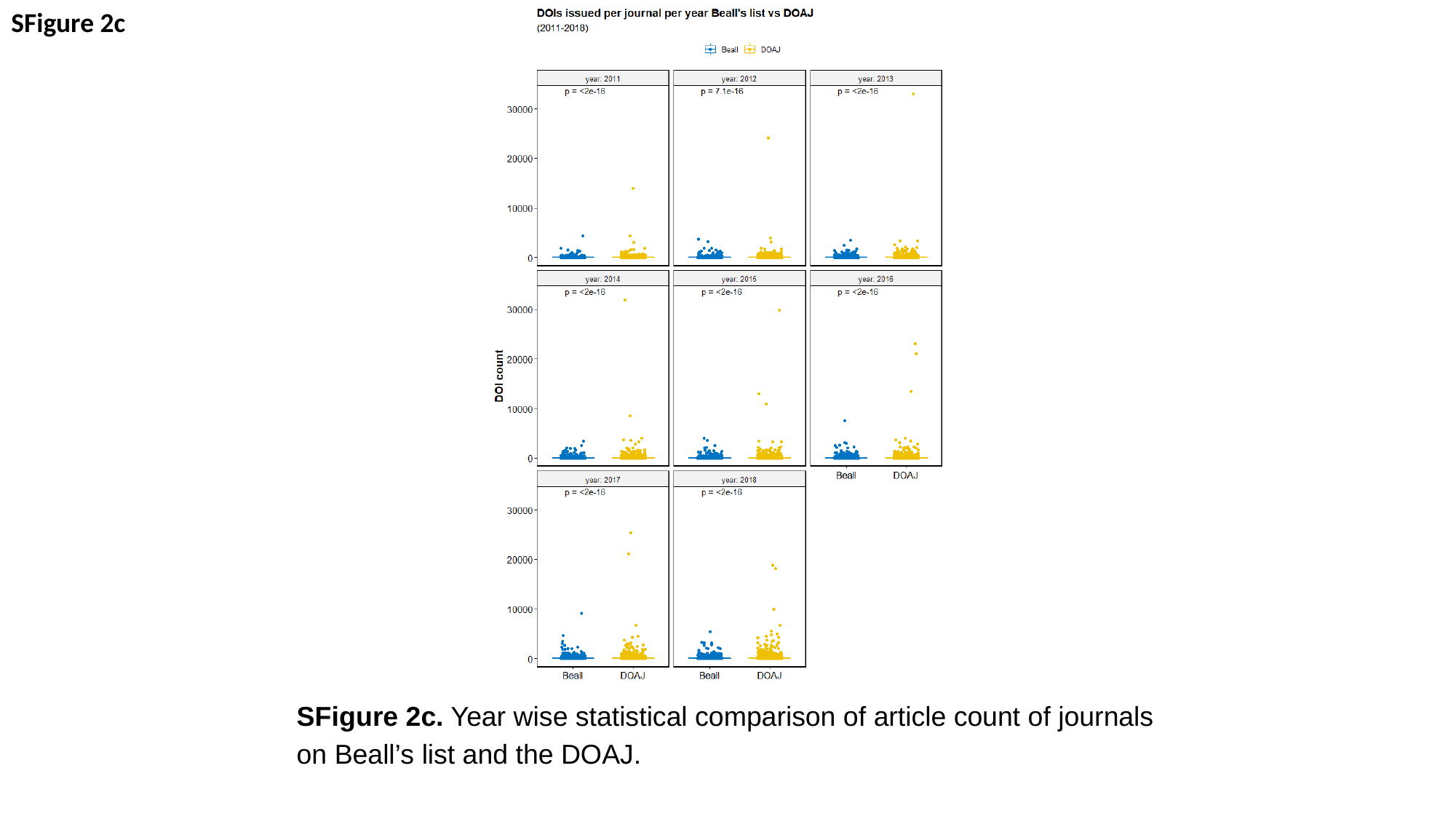

SFigure 2c
SFigure 2c. Year wise statistical comparison of article count of journals on Beall’s list and the DOAJ.

## Slide 8
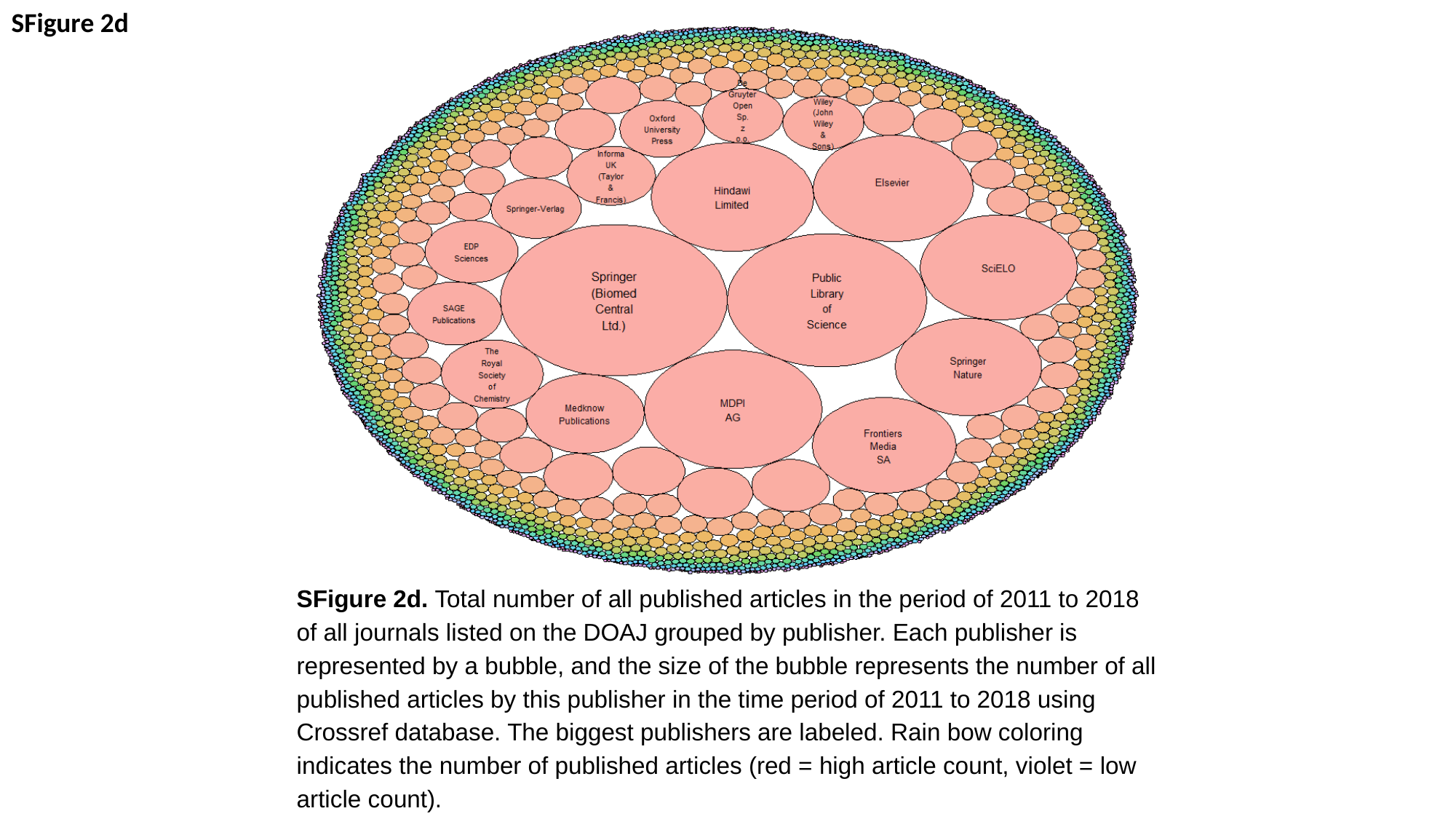

SFigure 2d
SFigure 2d. Total number of all published articles in the period of 2011 to 2018 of all journals listed on the DOAJ grouped by publisher. Each publisher is represented by a bubble, and the size of the bubble represents the number of all published articles by this publisher in the time period of 2011 to 2018 using Crossref database. The biggest publishers are labeled. Rain bow coloring indicates the number of published articles (red = high article count, violet = low article count).

## Slide 9
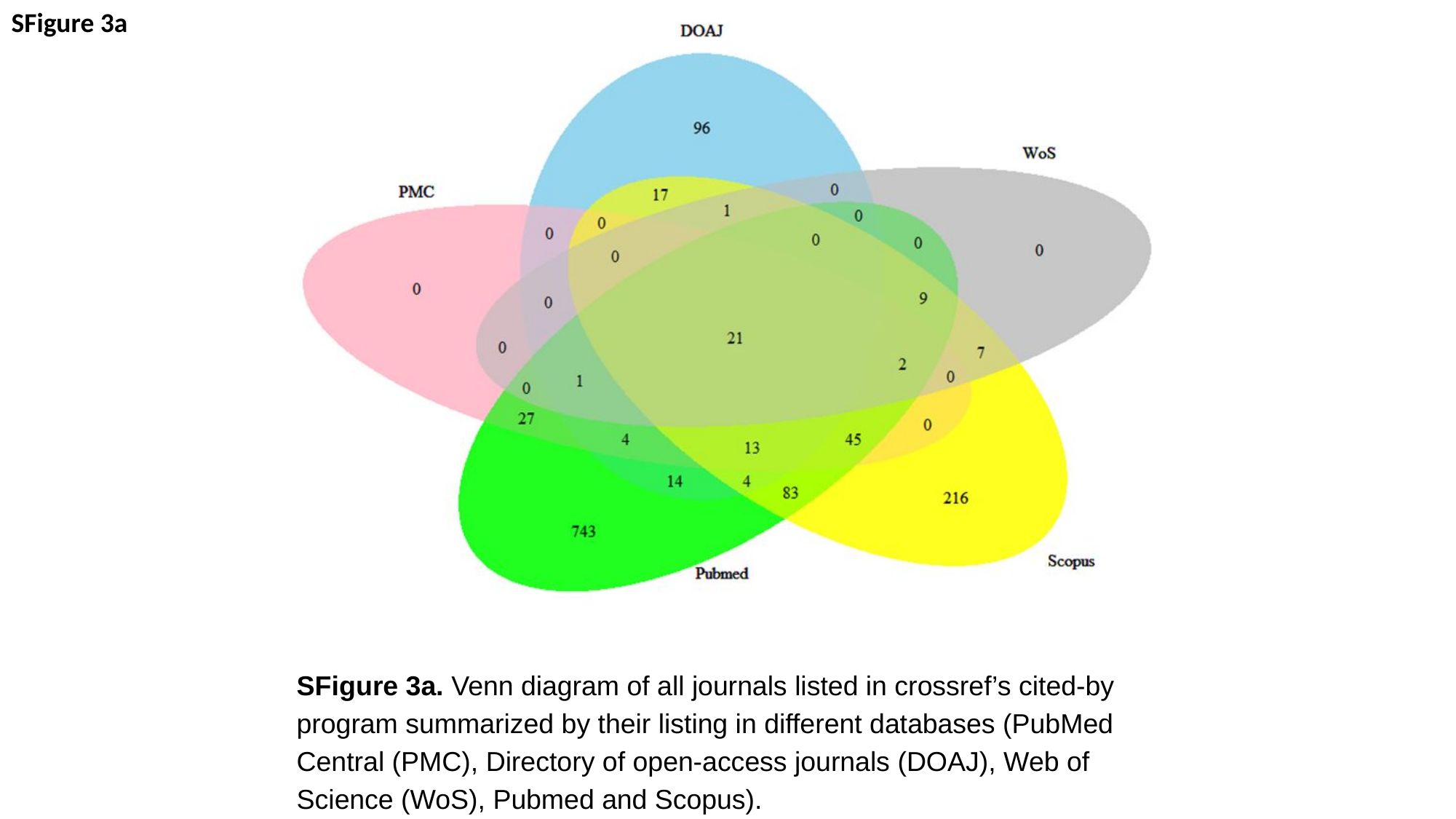

SFigure 3a
SFigure 3a. Venn diagram of all journals listed in crossref’s cited-by program summarized by their listing in different databases (PubMed Central (PMC), Directory of open-access journals (DOAJ), Web of Science (WoS), Pubmed and Scopus).

## Slide 10
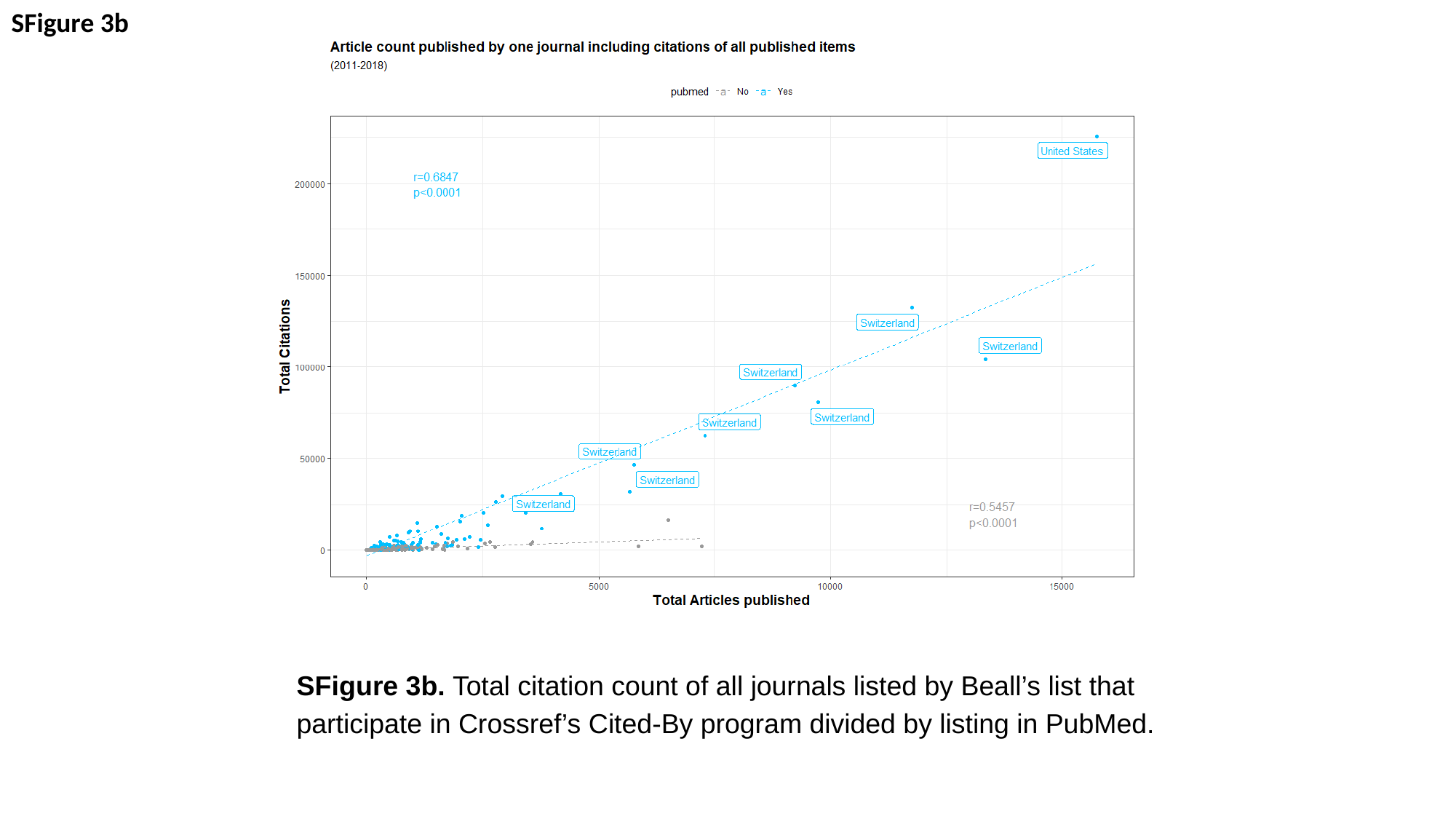

SFigure 3b
SFigure 3b. Total citation count of all journals listed by Beall’s list that participate in Crossref’s Cited-By program divided by listing in PubMed.

## Slide 11
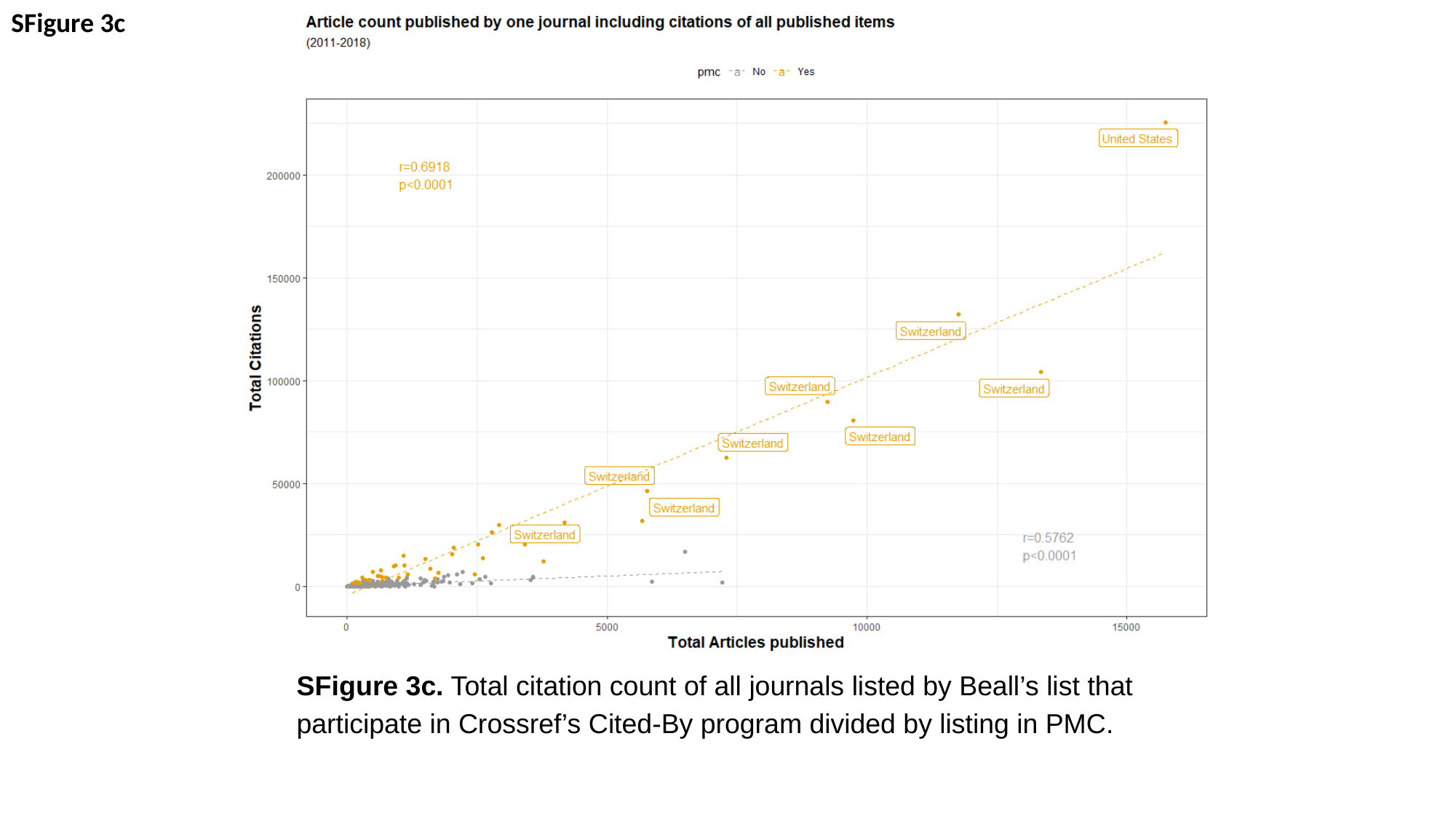

SFigure 3c
SFigure 3c. Total citation count of all journals listed by Beall’s list that participate in Crossref’s Cited-By program divided by listing in PMC.

## Slide 12
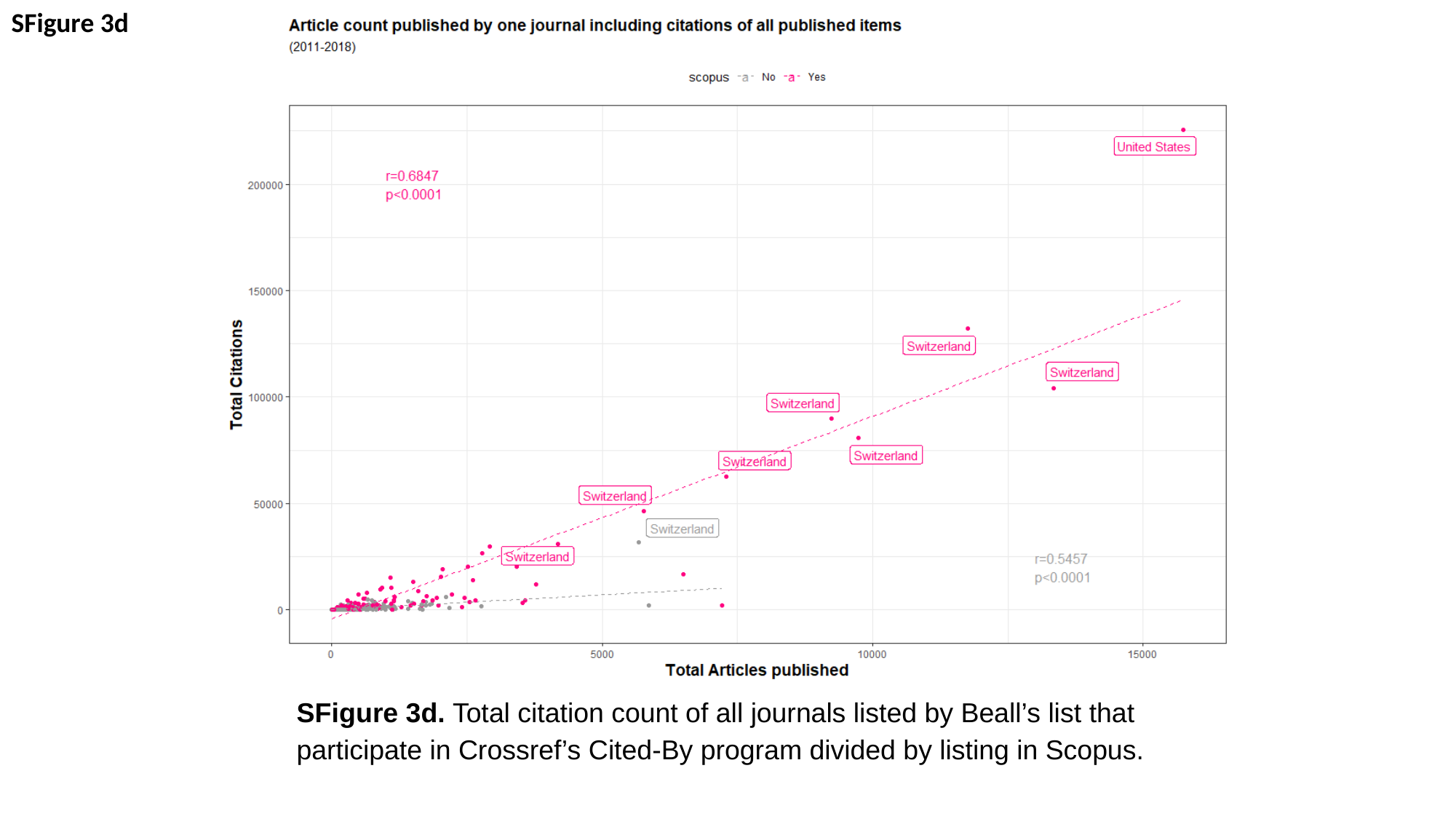

SFigure 3d
SFigure 3d. Total citation count of all journals listed by Beall’s list that participate in Crossref’s Cited-By program divided by listing in Scopus.

## Slide 13
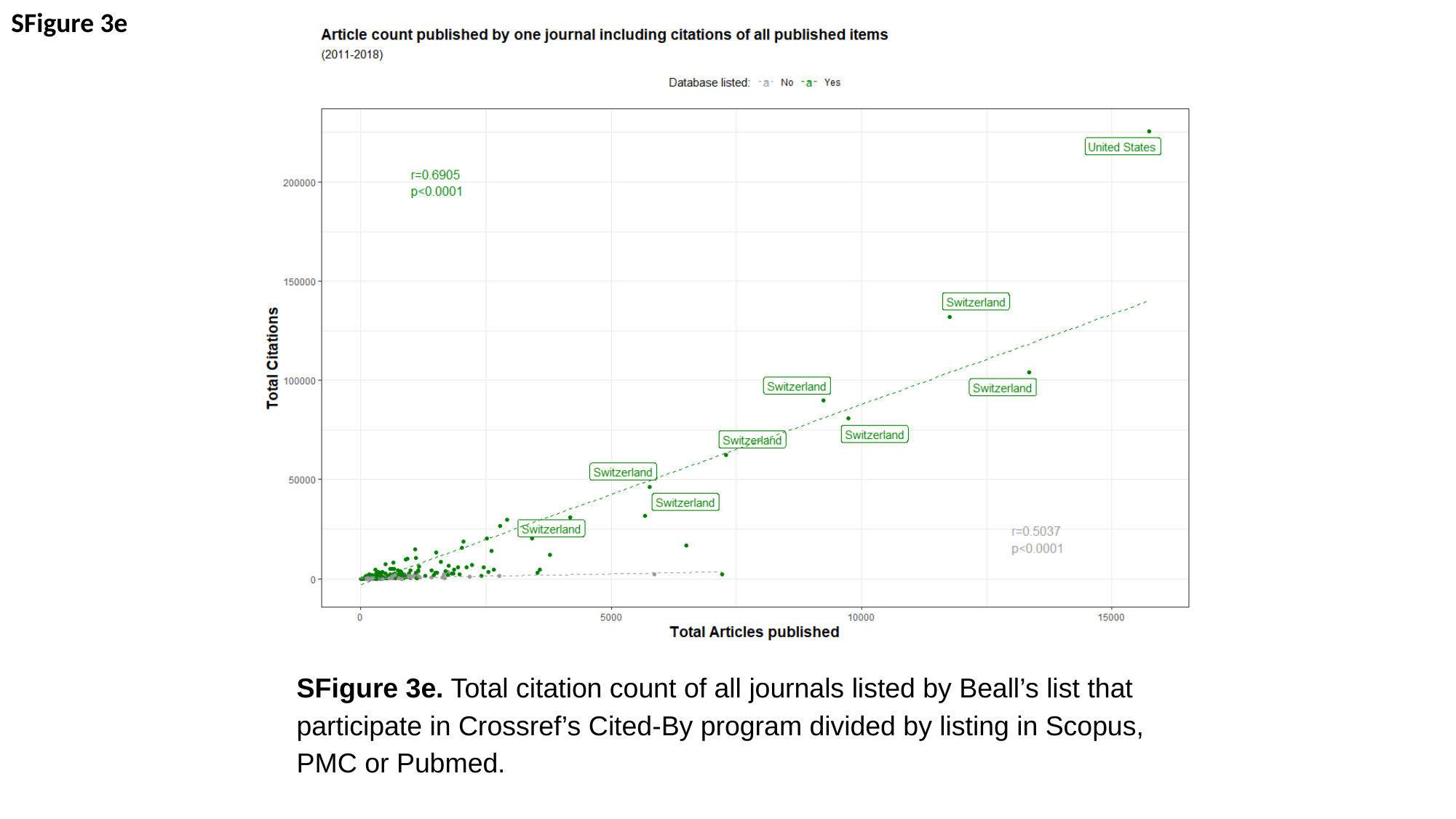

SFigure 3e
SFigure 3e. Total citation count of all journals listed by Beall’s list that participate in Crossref’s Cited-By program divided by listing in Scopus, PMC or Pubmed.

## Slide 14
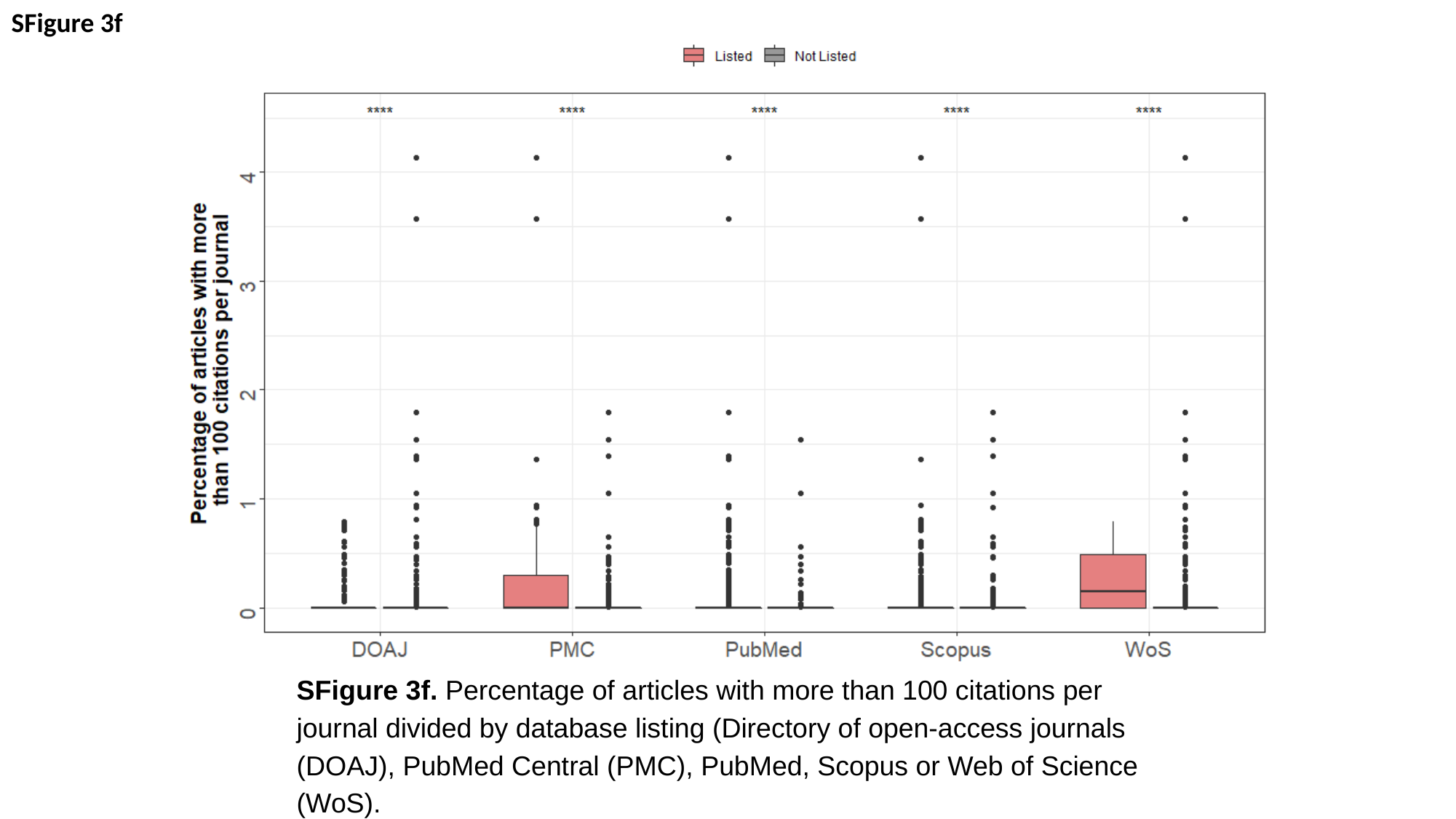

SFigure 3f
SFigure 3f. Percentage of articles with more than 100 citations per journal divided by database listing (Directory of open-access journals (DOAJ), PubMed Central (PMC), PubMed, Scopus or Web of Science (WoS).

## Slide 15
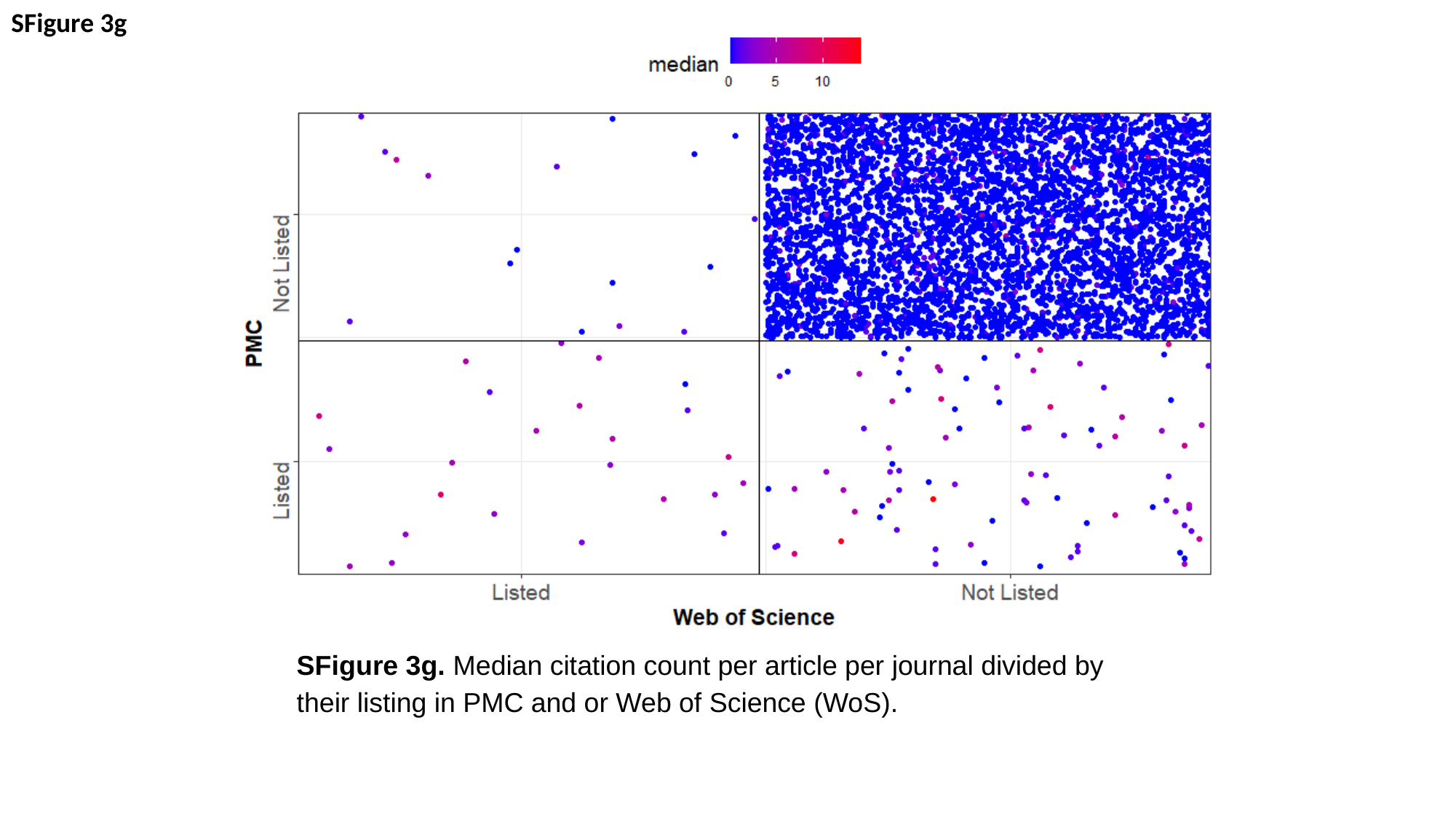

SFigure 3g
SFigure 3g. Median citation count per article per journal divided by their listing in PMC and or Web of Science (WoS).
