# Supplementary material for: Predatory journals: Perception, impact and use of Beall’s list by the scientific community–A bibliometric big data study
Source: PLoS One. 2023 Jul 7;18(7):e0287547. doi: 10.1371/journal.pone.0287547 (PMC10328228; doi:10.1371/journal.pone.0287547)
Supplement: S3 File — (DOCX) [file pone.0287547.s003.docx]

**Supplementary Tables**

**Table S1.** **Detailed analysis of title similarity of all 10354 unique ISSNs listed in Beall’s list searched in different databases.**

| Database (% of total) | Found in Database | Strict title similarity | Less strict title similarity | Levenshtein similarity | No title similarity | Manually Added | Total found |
| --- | --- | --- | --- | --- | --- | --- | --- |
| ISSN | 8671 (83.7) | 7135 (82.3) | 7405 (85.4) | 8266 (95.3) | 405 (4.7) | 0 (0.0) | 8266 (95.3) |
| Crossref | 5244 (50.7) | 4579 (87.3) | 4739 (90.4) | 5151 (98.2) | 93 (1.8) | 4 (0.1) | 5155 (98.3) |
| Scopus | 583 (5.6) | 512 (87.8) | 558 (95.7) | 568 (97.4) | 15 (2.6) | 2 (0.3) | 570 (97.8) |
| DOAJ | 226 (2.2) | 198 (87.6) | 201 (88.9) | 222 (98.2) | 4 (1.8) | 2 (0.9) | 224 (99.1) |
| PMC | 136 (1.32) | 126 (92.6) | 131 (96.3) | 135 (99.3) | 1 (0.7) | 0 (0.) | 135 (99.3) |
| Web of Science | 51 (0.5) | 47 (92.2) | 47 (92.2) | 49 (96.1) | 2 (3.9) | 1 (2.0) | 50 (98.0) |
| Pubmed | 1155 (11.2) | 980 (84.8) | 1046 (90.6) | 1129 (97.7) | 26 (2.3) | 11 (1.0) | 1139 (98.6) |

PMC = Pubmed Central; ISSN = International Standard Serial Number; DOAJ = Directory of Open-Access Journals;
Levenshtein cutoff values: ISSN (0.7), Crossref (0.5), Scopus (0.7), DOAJ (0.6), PMC (0.8), Web of Science (0.7), Pubmed (0.65).

ISSNs of journals listed on Beall’s list have been looked up if they were listed in the ISSN database, Crossref, Scopus, DOAJ, PMC, PubMed or Web of science and if the journal was the same in both databases. Therefore, the journal title in the specific database and on Beall’s list has been compared with three different methods: a highly strict similarity check where only articles have been removed; a less strict one where articles, publisher prefixes and other minor words have been removed and with the Levenshtein algorithm.

In the ISSN database have been 8671 ISSNs of journals on Beall’s list found. With the strict similarity check 7135 journals, with the less strict similarity check 7405 journals and with the Levenshtein similarity check (cutoff: 0.7) 8266 journals have successfully been found in the ISSN database. No title similarity was found in 405 cases and 0 journals were manually added. In total 8266 journals on Beall’s list were found in the ISSN database after journal title comparison with three different algorithm.

**Table S2a.** **Detailed descriptive statistics of articles published by journals listed in Crossref and on Beall’s list divided by years.**

| **# of journals analysed** | **Min** | **Max** | **Median** | **Mean** | **IQR** |
| --- | --- | --- | --- | --- | --- |
| **Year 2011** | | | | | |
| 945 | 1 | 4412 | 18 | 59.57 | 40 |
| **Year 2012** | | | | | |
| 1342 | 1 | 3739 | 24 | 63.89 | 45 |
| **Year 2013** | | | | | |
| 1820 | 1 | 3501 | 21 | 59.85 | 40 |
| **Year 2014** | | | | | |
| 2280 | 1 | 3411 | 19 | 56 | 38 |
| **Year 2015** | | | | | |
| 2792 | 1 | 3974 | 19 | 51.44 | 34 |
| **Year 2016** | | | | | |
| 3275 | 1 | 7533 | 18 | 53.66 | 34 |
| **Year 2017** | | | | | |
| 3494 | 1 | 9141 | 17 | 56.54 | 34 |
| **Year 2018** | | | | | |
| 3393 | 1 | 5394 | 14 | 49.81 | 30 |

IQR = interquartile range

DOI information (as surrogate for published articles) stored in the Crossref database have been linked with the journals listed on Beall’s list and analyzed for article count over the time period of 2011 to 2018.

In the year 2011, 945 journals listed on Beall’s list published at least one article, whereas the minimum article count per journal was 1 and the maximum 4412. The median was 18 articles per journal, the mean 59.57 and the IQR 40 in the year 2011.

**Table S2b. Results of the Kruskal-Wallis-Test with pairwise comparisons using Wilcoxon rank sum test with continuity correction and post-hoc correction using Bonferroni’s post-hoc correction.**

|  | Kruskal-Wallis-Test: X² = 132.77, df = 7, p-value **< 2.2e-16** | | | | | | |
| --- | --- | --- | --- | --- | --- | --- | --- |
| Pairwise comparisons using Wilcoxon rank sum test with continuity correction and Bonferroni’s post-hoc correction: | | | | | | | |
|  | 2011 | 2012 | 2013 | 2014 | 2015 | 2016 | 2017 |
| 2012 | **0.00414** | - | - | - | - | - | - |
| 2013 | 0.34057 | 1.00000 | - | - | - | - | - |
| 2014 | 1.00000 | **0.02620** | 1.00000 | - | - | - | - |
| 2015 | 1.00000 | **0.00325** | 1.00000 | 1.00000 | - | - | - |
| 2016 | 1.00000 | **0.00049** | 0.42518 | 1.00000 | 1.00000 | - | - |
| 2017 | 1.00000 | **3.6e-07** | **0.00145** | 0.33709 | 0.44945 | 1.00000 | - |
| 2018 | **0.02890** | **< 2e-16** | **1.5e-14** | **1.6e-10** | **1.3e-11** | **3.6e-11** | **1.3e-05** |

P values lower than 0.05 (and therefore considered statistically significant) are shown in bold.

**Table S3. Analysis of changes in the yearly issued DOI count for all journals listed on Beall’s list over the period of 2011 to 2018.**

| **Increase** | | | | | | | | |
| --- | --- | --- | --- | --- | --- | --- | --- | --- |
| Time | 0 year | 1 year | 2 years | 3 years | 4 years | 5 years | 6 years | 7 years |
| Journals | 112 | 1246 | 1344 | 1118 | 555 | 177 | 26 | 4 |
| **Decrase** | | | | | | | | |
| Time | 0 year | 1 year | 2 years | 3 years | 4 years | 5 years | 6 years | 7 years |
| Journals | 678 | 1356 | 1165 | 769 | 430 | 157 | 19 | 8 |
| **No change** | | | | | | | | |
| Time | 0 year | 1 year | 2 years | 3 years | 4 years | 5 years | 6 years | 7 years |
| Journals | 864 | 592 | 593 | 669 | 699 | 777 | 353 | 35 |

DOI information (as surrogate for published articles) stored in the Crossref database have been linked with the journals listed on Beall’s list and analyzed for the change in article count within the years of 2011 to 2018.

4 journals could increase their article count per year every year for 7 years, followed by 26 journals for 6 years. 8 journals had a decrease in their article count per year every year for 7 years, followed by 19 for 6 years.

**Table S4a.** **Descriptive analysis of the article counts from all journals listed in the DOAJ and registered in Crossref per year between 2011 and 2018.**

| **# of journals analysed** | **min** | **max** | **median** | **mean** | **IQR** |
| --- | --- | --- | --- | --- | --- |
| **Year 2011** | | | | | |
| 3521 | 1 | 14047 | 29 | 62 | 41 |
| **Year 2012** | | | | | |
| 4177 | 1 | 24111 | 30 | 67 | 43 |
| **Year 2013** | | | | | |
| 4849 | 1 | 32992 | 29 | 69 | 41 |
| **Year 2014** | | | | | |
| 5615 | 1 | 31880 | 29 | 70 | 39 |
| **Year 2015** | | | | | |
| 6309 | 1 | 29815 | 29 | 71 | 41 |
| **Year 2016** | | | | | |
| 6779 | 1 | 23022 | 30 | 72 | 40 |
| **Year 2017** | | | | | |
| 7129 | 1 | 25341 | 30 | 73 | 40 |
| **Year 2018** | | | | | |
| 7029 | 1 | 18832 | 31 | 81 | 42 |

IQR = interquartile range

DOI information (as surrogate for published articles) stored in the Crossref database have been linked with the journals listed in the DOAJ and analyzed for article count over the time period of 2011 to 2018.

In the year 2011, 3521 journals listed in the DOAJ published at least one article, whereas the minimum article count per journal was 1 and the maximum 14047. The median was 29 articles per journal, the mean 62 and the IQR 41 in the year 2011.

**Table S4b. Results of the Kruskal-Wallis-Test with pairwise comparisons using Wilcoxon rank sum test with continuity correction and post-hoc correction using Bonferroni’s post-hoc correction.**

|  | Kruskal-Wallis-Test: X² = 34.543, df = 7, p-value = **1.363e-05** | | | | | | |
| --- | --- | --- | --- | --- | --- | --- | --- |
| Pairwise comparisons using Wilcoxon rank sum test with continuity correction and Bonferroni’s post-hoc correction: | | | | | | | |
|  | 2011 | 2012 | 2013 | 2014 | 2015 | 2016 | 2017 |
| 2012 | 1.0000 | - | - | - | - | - | - |
| 2013 | 1.0000 | 1.0000 | - | - | - | - | - |
| 2014 | 1.0000 | 1.0000 | 1.0000 | - | - | - | - |
| 2015 | 1.0000 | 1.0000 | 1.0000 | 1.0000 | - | - | - |
| 2016 | **0.0302** | 1.0000 | 0.2826 | 0.2421 | 1.0000 | - | - |
| 2017 | **0.0134** | 1.0000 | 0.1343 | 0.1066 | 0.8569 | 1.0000 | - |
| 2018 | **0.0012** | 0.2629 | **0.0125** | **0.0088** | 0.1095 | 1.0000 | 1.0000 |

P values lower than 0.05 (and therefore considered statistically significant) are shown in bold.

**Table S5.** **Analysis of changes in the yearly issued DOI count for all journals listed in the DOAJ over the period of 2011 to 2018.**

| **Increase** | | | | | | | | |
| --- | --- | --- | --- | --- | --- | --- | --- | --- |
| Time | 0 year | 1 year | 2 years | 3 years | 4 years | 5 years | 6 years | 7 years |
| # of journals | 120 | 966 | 1796 | 2219 | 1701 | 787 | 235 | 52 |
| **Decrease** | | | | | | | | |
| Time | 0 year | 1 year | 2 years | 3 years | 4 years | 5 years | 6 years | 7 years |
| # of journals | 986 | 1690 | 1890 | 1889 | 1055 | 320 | 46 | 0 |
| **No change** | | | | | | | | |
| Time | 0 year | 1 year | 2 years | 3 years | 4 years | 5 years | 6 years | 7 years |
| # of journals | 2860 | 1421 | 1076 | 896 | 660 | 581 | 309 | 73 |

DOI information (as surrogate for published articles) stored in the Crossref database have been linked with the journals listed in the DOAJ and analyzed for the change in article count within the years of 2011 to 2018.

52 journals could increase their article count per year every year for 7 years, followed by 235 journals for 6 years. 0 journals had a decrease in their article count per year every year for 7 years, followed by 46 for 6 years.

**Table S6. Journals listed on Beall’s list and their participation in the Cited-by program by Crossref according to their country information, as provided by the ISSN database.**

|  | **Participation in the cited-by program?** | |  | **Participation in the cited-by program?** | |
| --- | --- | --- | --- | --- | --- |
| **Country** | **Yes (%)** | **No (%)** | **Country** | **Yes (%)** | **No (%)** |
| Algeria | 0 (0.0) | 1 (0.0) | Kazakhstan | 0 (0.0) | 2 (0.0) |
| Argentina | 0 (0.0) | 1 (0.0) | Kenya | 0 (0.0) | 2 (0.0) |
| Australia | 0 (0.0) | 29 (0.7) | Republic of Korea | 0 (0.0) | 31 (0.7) |
| Austria | 0 (0.0) | 4 (0.1) | Lithuania | 4 (0.5) | 0 (0.0) |
| Azerbaijan | 0 (0.0) | 4 (0.1) | Macedonia | 0 (0.0) | 8 (0.2) |
| Bangladesh | 0 (0.0) | 13 (0.3) | Malaysia | 0 (0.0) | 4 (0.1) |
| Belgium | 0 (0.0) | 2 (0.0) | Mauritius | 0 (0.0) | 1 (0.0) |
| Brazil | 0 (0.0) | 1 (0.0) | Mexico | 0 (0.0) | 4 (0.1) |
| Bulgaria | 3 (0.4) | 22 (0.5) | Morocco | 0 (0.0) | 2 (0.0) |
| Cameroon | 0 (0.0) | 1 (0.0) | Nepal | 0 (0.0) | 2 (0.0) |
| Canada | 10 (1.2) | 182 (4.2) | Netherlands | 3 (0.4) | 13 (0.3) |
| China | 0 (0.0) | 1 (0.0) | New Zealand | 1 (0.1) | 0 (0.0) |
| Croatia | 0 (0.0) | 3 (0.0) | Nigeria | 33 (4.1) | 71 (1.6) |
| Cyprus | 0 (0.0) | 3 (0.0) | Pakistan | 62 (7.6) | 197 (4.5) |
| Czech Republic | 0 (0.0) | 6 (0.1) | Philippines | 0 (0.0) | 4 (0.1) |
| Denmark | 0 (0.0) | 1 (0.0) | Qatar | 0 (0.0) | 1 (0.0) |
| Egypt | 4 (0.5) | 14 (0.3) | Romania | 1 (0.1) | 7 (0.1) |
| Estonia | 0 (0.0) | 1 (0.0) | Russian Federation | 0 (0.0) | 2 (0.0) |
| Ethiopia | 0 (0.0) | 8 (0.2) | Serbia | 2 (0.2) | 1 (0.0) |
| France | 0 (0.0) | 1 (0.0) | Singapore | 14 (1.7) | 19 0.4) |
| Germany | 0 (0.0) | 13 (0.3) | Slovakia | 1 (0.1) | 1 (0.0) |
| Greece | 0 (0.0) | 15 (0.3) | Spain | 0 (0.0) | 15 (0.3) |
| Guyana | 0 (0.0) | 2 (0.0) | Switzerland | 68 (8.4) | 5 (0.1) |
| Hong Kong S.A.R., China | 1 (0.1) | 76 (1.8) | Taiwan, Province of China | 2 (0.2) | 0 (0.0) |
| Hungary | 0 (0.0) | 1 (0.0) | Turkey | 4 (0.5) | 23 (0.5) |
| India | 101 (12.4) | 1049 (24.2) | Ukraine | 18 (2.2) | 0 (0.0) |
| Indonesia | 0 (0.0) | 5 (0.1) | United Arab Emirates | 4 (0.5) | 75 (1.7) |
| International organization | 2 (0.2) | 2 (0.0) | United Kingdom | 44 (5.3) | 273 (6.3) |
| Islamic Republic of Iran | 30 (3.7) | 6 (0.1) | United States | 359 (44.2) | 1817 (41.9) |
| Iraq | 0 (0.0) | 1 (0.0) | unknown | 36 (4.4) | 273 (6.3) |
| Italy | 3 (0.4) | 3 (0.0) | Vietnam | 0 (0.0) | 1 (0.0) |
| Japan | 2 (0.2) | 1 (0.0) | Virgin Islands, British | 0 (0.0) | 2 (0.0) |
| Jordan | 0 (0.0) | 5 (0.1) | **Total count** | 812 (100.0) | 4333 (100.0) |

Journals listed on Beall’s list have been linked with crossref’s database and with the country information of the ISSN database.

The majority of participants of the cited-by program came from the United states (44.2%), followed by india (12.4%) and Switzerland (8.4%).

**Table S7.** **Journals listed on Beall’s list and their participation in the Cited-by program of Crossref divided by their listing in different databases.**

| Listed in **PMC**? | Participation in the Cited-By Program? | | CI 95% | | OR |
| --- | --- | --- | --- | --- | --- |
|  | Yes | No | lower | upper |  |
| Yes | 73 | 45 | 6.3 | 14.1 | 9.4 |
| No | 739 | 4288 |  |  |  |
| Listed in **DOAJ**? | Participation in the Cited-By Program? | | CI 95% | | OR |
|  | Yes | No | lower | upper |  |
| Yes | 83 | 83 | 4.2 | 8.1 | 5.8 |
| No | 729 | 4250 |  |  |  |
| Listed in **Scopus**? | Participation in the Cited-By Program? | | CI 95% | | OR |
|  | Yes | No | lower | upper |  |
| Yes | 166 | 646 | 3.0 | 4.7 | 3.8 |
| No | 277 | 4056 |  |  |  |
| Listed in **Pubmed**? | Participation in the Cited-By Program? | | CI 95% | | OR |
|  | Yes | No | lower | upper |  |
| Yes | 232 | 646 | 1.6 | 2.3 | 1.9 |
| No | 580 | 4056 |  |  |  |
| Listed in **WoS**? | Participation in the Cited-By Program? | | CI 95% | | OR |
|  | Yes | No | lower | upper |  |
| Yes | 29 | 15 | 5.5 | 21.5 | 10.7 |
| No | 4318 | 783 |  |  |  |

PMC = PubMed Central; DOAJ = Directory of Open Access Journals; WoS = Web of Science; CI = Confidence Interval; OR = Odds Ratio

Information if journals were participants of the cited-by program or not have been linked with the information if the journal is listed in PMC, DOAJ, Scopus, PubMed or Web of Science.

Journals that have been listed in Web of Science had a 10.7 higher likelihood of being participant of the cited-by program, followed by journals listed in PMC with a likelihood of 9.4.
