# Supplementary material for: Predatory journals: Perception, impact and use of Beall’s list by the scientific community–A bibliometric big data study
Source: PLoS One. 2023 Jul 7;18(7):e0287547. doi: 10.1371/journal.pone.0287547 (PMC10328228; doi:10.1371/journal.pone.0287547)
Supplement: Attachment — Submitted filename: Rebuttal_letter_PONE-D-22-08372_v11152022.pdf [file pone.0287547.s005.pdf]

PONE-D-22-08372

Dear Dr. Lopes, 15 November 2022

we thank the reviewers for their valuable comments and the Editorial Board for the consideration of our work for possible publication in PLOS One.

The following changes have been made according to the suggestions of all reviewers:

Reviewer #1: please try to incorporate the comments to give more sound for the reader/researcher when the manuscript published. The author must focus on the data explanation and he/she must use appropriate model for data analysis.

Answer #1: We thank the reviewer for his/her valuable suggestions and made the following changes to the manuscript according to the reviewer's suggestions:

1) We further streamlined the introduction to give a more explanatory overview on why predatory journals are a serious problem for the scientific community.
2) The discussion section has been updated to put our results in a clearer picture according to the current literature. Additionally, we rewrote the limitations section to provide readers with guidance on the interpretation of our findings and report.
3) We further included some explanatory sentences within the results section to provide focus on data explanation and context on the models of data analysis used.
4) Concerning the use of appropriate models for data analysis, we introduced several statistical analyses and present their results. A statistical section has been added to the methods section. Furthermore, Table S2b, Table S4b, SFig 2c were added, and Fig 4a-b, Fig 5a-c and SFig 3b-f were updated. (Figure 2 has been split into Figure 2 and Figure 3. Figure 3 has been split into Figure 4 and Figure 5. Both for better readability).

5) The title has been changed from “Perception, impact and use of Beall’s list by the scientific community” to “Predatory journals: Perception, impact and use of Beall’s list by the scientific community – a bibliometric big data study”. Short title has been included.

Reviewer #2.1:

1. There are a huge number of statistics in this manuscript; but the background information is not sufficiently provided. In other words, the authors need to clarify why publishing in predatory journals is problematic. If so, how the scientific community should be aware of this problem. Basically, for what reason(s) these journals were created? Is that a kind of dirty business or it is in response to some demand.

The readers of articles of this kind, would like to see some opinions on this issue. Providing statistics in not enough.

Answer #2.1: We thank the reviewer for this important comment. We agree with the reviewer that it is important to provide more context on why predatory journals are problematic and why they exist. Predatory journals emerged in the wake of the open access (OA) publishing model, where authors pay a fee for getting their work published and made available to the public. Quality publishing is time-consuming and costly, and some journals decided to put revenue first and quality second, set out to attract submission of as many articles as possible independent of their quality and scientific merit.

From our perspective, there are several problems with publishing in a predatory journal. These include, but are not limited to the fact that the lack of peer-review bears the risk of publishing data that are not fully scientifically sound. Peer review, i.e. the evaluation of reports by one or more scientists with similar competencies, is an important instrument of self-regulation that helps to maintain quality standards, avoid plagiarism, and improve the quality of scientific reporting. A negative peer review should prompt journals to ask authors to improve their report or to reject the paper. This does not happen with predatory journals. Secondly, predatory journals have low or no standards with respect to the reporting of conflicts of interests, which

results in the risk that such conflicts are not or not fully made transparent. Thirdly, many predatory journals exist for only a short period of time, after which their content and published reports are no longer available, resulting in loss of information.

In response to your question and suggestion, we have now included additional wording on predatory journals and why they exist and why they are problematic:

There are several problems with publishing in a predatory journal. These include, but are not limited to the fact that the lack of peer-review bears the risk of publishing data that are not fully scientifically sound. Peer review, i.e. the evaluation of reports by one or more scientists with similar competencies, is an important instrument of self-regulation that helps to maintain quality standards, avoid plagiarism, and improve the quality of scientific reporting. A negative peer review should prompt journals to ask authors to improve their report or to reject the paper. This does not happen with predatory journals. Secondly, predatory journals have low or no standards with respect to the reporting of conflicts of interests, which results in the risk that such conflicts are not or not fully made transparent. Thirdly, many predatory journals exist for only a short period of time, after which their content and published reports are no longer available, resulting in loss of information.[2]

Reviewer #2.2: 2. The authors report that some of the predatory journals on the Beall's list are also listed in quality-driven databases such as PubMed, CrossRef, etc. The authors need to explain how these predatory journals' names were introduced into the quality-driven databases. Is that through referencing and citation by published articles? or by other means?

Answer #2.2: There are two types of databases available for science: Quality-driven database and others. In quality-driven databases the journal must meet some content criteria that go beyond formal criteria. The most rigorous criteria - in terms of form and content - were set by Web of science followed by MEDLINE. It is important to know that one does not necessarily have to be listed in MEDLINE to appear in a Pubmed search. Pubmed has also a) changed its criteria after the first large study and b) also issued a statement! (https://grants.nih.gov/grants/guide/notice-files/not-od-18-011.html) In our study, we also show quite clearly that the number of journals that were listed in Beall's list appeared relatively rarely in Web of Science in contrast to databases that do not do a content check but only formal background check (e.g.

Crossref). Journals apply for inclusion in a database and have to meet only formal criteria (e.g. crossref) or also content criteria (e.g. Web of science).

We inserted the following phrase to the discussion to clarify this issue:

“Another important criterion of the scientific quality of a journal is its listing in databases. Journals can apply for inclusion by a specific database. Since different databases have different inclusion criteria, it makes a significant difference in which database a journal is listed. Web of Science has the most rigorous criteria for inclusion in their database, and the number of Beall’s journals listed there was the lowest. On the other side, the highest number of journals on Beall’s list was listed in crossref, which performs only a formal background check. “

The methodological differentiation between quality-driven and non-quality-driven is now stated in the methods section:

“Databases were categorized as quality-driven or non-quality-driven databases depending on their journal inclusion criteria. If journals only had to fulfil formal criteria to be included in the database, the database was categorized as non-quality-driven (e.g. ISSN database, Crossref). If as database performs substantial background checks prior to inclusion of a journal, the database was categorized as quality-driven (e.g. WoS, PMC, Pubmed, Scopus, DOAJ).“

Reviewer #2.3: 3. I think it is very important to define a “predatory journal’. It is hard to believe that the publishers that publish peer-reviewed articles are also publishing non-peer-reviewed articles. For example, Frontiers Media and OMICS Publishing Group that publish high impact factor open access journals are also publishing predatory journals? If it is true, needs more clarification. How these two sorts of journals i.e. true scientific journals and predatory journals, were mixed up.

Answer #2.3: The definition of a “predatory journal” is rather straightforward and was reviewed by several authors in more detail including our group (Richtig et al. JEADV 2018; Beall JOSPT 2017). Briefly, a predatory journal accepts articles for publication, along with authors fees, without checks for quality, plagiarism, or ethical

approval. The main challenge and the real underlying problem are how to identify “predatory journals”. In essence, whether a journal is “predatory” or not depends on its aims, intentions, and processes, which often are not made known to the general public. With non-predatory journals, peer review is the rule and publication of a paper without peer-review is a mistake that should be detected and avoided. With predatory journals, absence of peer-review or very poor review are systematic and intended (or at tolerated without intention to change this practice). Without precise knowledge of a journal’s internal structures and practices, one cannot make a statement about whether a journal is "predatory" (Discussed in Richtig et al. JEADV 2018). Also, a "predatory journal", over time, can become a non predatory one. A new open access journal with an inexperienced editor and publishing team may be viewed as "predatory" because of how it works, but has a genuine interest in becoming a “good” journal, and does so over time. Many lists of “predatory” journals readily include journals but rarely drop them (compare Figure 2 Richtig et al. JEADV 2018). Taken together, the problem is complex and was not the focus of our study, which aimed to assess how a very prominent list of predatory journals (Beall's list was the most prestigious and widely discussed) was used by the scientific community.

To address the reviewer’s feedback, we updated the section on limitations:

“Firstly, Beall’s list has been criticized for its flawed methodology and should no longer be used to identify predatory journals. The goal of the present study was not to classify journals as predatory or non-predatory, but rather to investigate the impact of Beall’s list on scientific publishing over the time period it was online.“

Editor comment #3.1:

1. Please ensure that your manuscript meets PLOS ONE's style requirements, including those for file naming. The PLOS ONE style templates can be found at https://journals.plos.org/plosone/s/file?id=wjVg/PLOSOne_formatting_sample_main_body.pdf and

https://journals.plos.org/plosone/s/file?id=ba62/PLOSOne_formatting_sample_title_authors_affiliations.pdf

Answer #3.1: We thank the Editor for this advice. We have reviewed and adopted our manuscript to meet PLOS ONE's style requirements.

Editor comment #3.2:

We note that you have indicated that data from this study are available upon request. PLOS only allows data to be available upon request if there are legal or ethical restrictions on sharing data publicly. For more information on unacceptable data access restrictions, please see http://journals.plos.org/plosone/s/data-availability#loc-unacceptable-data-access-restrictions.

In your revised cover letter, please address the following prompts:

a) If there are ethical or legal restrictions on sharing a de-identified data set, please explain them in detail (e.g., data contain potentially sensitive information, data are owned by a third-party organization, etc.) and who has imposed them (e.g., an ethics committee). Please also provide contact information for a data access committee, ethics committee, or other institutional body to which data requests may be sent.

b) If there are no restrictions, please upload the minimal anonymized data set necessary to replicate your study findings as either Supporting Information files or to a stable, public repository and provide us with the relevant URLs, DOIs, or accession numbers. For a list of acceptable repositories, please see http://journals.plos.org/plosone/s/data-availability#loc-recommended-repositories.

We will update your Data Availability statement on your behalf to reflect the information you provide

Answer #3.2: Although all lists that are needed to reproduce this study can be downloaded by using wayback.org, we made a zip file with all text files to reproduce this study. This contains all lists from the methods section: “First, complete lists of journals indexed by the following databases were obtained: Scopus (downloaded from Elsevier’s Scopus database; https://www.scopus.com; 18.06.2018), Web of Science (WoS; downloaded from Clarivate Analytics; Journal Citation Reports 2017; https://jcr.clarivate.com; 12.04.2018), the Directory of Open-Access Journals (DOAJ;

https://doaj.org/csv; 11.04.2018); PubMed (acquired from the U.S. National Institutes of Health (NIH; ftp://ftp.ncbi.nih.gov/pubmed/J_Medline.txt;_15.12.2018), and the U.S. National Library of Medicine's list of PubMed Central (PMC) participating journals (https://www.ncbi.nlm.nih.gov/pmc/journals; 15.12.2018)".

1.) bealls_journals.csv (the used version of Beall's List from the homepage of Beall)(journals)
2.) bealls_publisher.csv (the used version of Beall's List from the homepage of Beall)(publisher)
3.) predJournals_final4_15092018_cleaned_double.csv
4.) publisher_only_from14082017.csv
5.) PubMed: J_Medline_20181215.txt
6.) PMC: jlist_20181215.csv
7.) Web of Science: Journal_Citation_report_12042018.csv
8.) DOAJ: doaj_20180411_1930_utf8.csv
9.) Scopus: ext_list_April_2018_2017_Metrics_18062018_cleaned.csv
10.) Crossref: All Dois found published by journals on bealls list: all_dois.txt

The whole dataset contains about 250 GB of plain text and it would, therefore, be too large for complete upload. However, with the dataset provided it should be possible to reproduce the study where static databases have been used (except crossref whereas the citation data has been obtained and should be continuously updated by crossref itself).
