# Supplementary material for: Predatory journals: Perception, impact and use of Beall’s list by the scientific community–A bibliometric big data study
Source: PLoS One. 2023 Jul 7;18(7):e0287547. doi: 10.1371/journal.pone.0287547 (PMC10328228; doi:10.1371/journal.pone.0287547)
Supplement: Attachment — Submitted filename: Rebuttal_letter_PONE-D-22-08372R2_final_2.pdf [file pone.0287547.s007.pdf]

PONE-D-22-08372R3

Dear Dr. Lopes, 23th May 2023

we thank the reviewers for their valuable comments and the Editorial Board for the consideration of our work for possible publication in PLOS One.

The following changes have been made according to the suggestions of all reviewers:

Academic Editor:

Due to its controversial potential, the manuscript needs to be revised by clarifying some aspects. Please see bellow.

"The manuscript's conclusions are overstated in some cases. As an observational study the authors need to apply caution in inferring causation. We don't know what would have happened to the titles in Beall's list had they not been included, so we can't really include anything about the direct impact of inclusion in the list.

Answer #1: Thanks for raising this point, it is well taken. We revised the manuscript accordingly, bringing your suggestions on board.

Revisions are required to address, as follows:

Comment #1.1: Line 47: "Beall’s list, for the scientific community, appears not to be crucial for publishing in journals or for citing their content."

Answer #1.1: Thanks for the suggestion. We reworded the sentence: „It seems that the importance of Beall’s list for the scientific community is overestimated.“

Comment #1.2: Line 345: "Beall’s list did not significantly impact the Open-Access movement." - needs to be toned down - needs to report result here (that articles list in Beall's list show similar publishing patterns to those not included in the list)

Answer #1.2: We thank the reviewer for his comment and changed the sentence from „Beall's list did not significantly impact the Open-Access movement" to „ Articles published in journals in Beall's list show similar publishing patterns to those in journals not included in the list, suggesting that Beall's list had no major influence on the Open-Access movement".
The comparison of Beall's list with the DOAJ starts with Figure 2, where there is a direct comparison of both publication numbers, followed by subsequent analysis of both lists.

Comment #1.3: Line 548: "Although there was and is a substantial discussion around Beall's list and predatory journals, this seems to have had little impact on scientific publishing itself"

Answer #1.3: Line 548 „Although there was and is a substantial discussion around Beall's list and predatory journals, this seems to have had little impact on scientific publishing itself" has been changed to „Although Beall's list received broad attention by the scientific community, but it seems its impact on scientific publishing may be overestimated."

Comment #1.4: The section 'Beall's list did not significantly impact the Open-Access movement' talks about journals being successful and unsuccessful. This language is ambiguous (a journal could be successful by reducing its published output, but increasing standards) and needs to be refined.

Answer #1.4: We agree with this comment and reworded this section accordingly. Specifically, the section now reads: "When the journals' annual output was more closely analysed over the past seven years, 52 journals (0.7%) increased the number of published articles from year to year (median: 3), and no journal had a continuous decrease over the entire seven year period. When journals were grouped by their publishers, 104 of 2,296 (4.5%) publishers (287 journals) had a continuous increase in published articles over the six (or seven) year period. Within the DOAJ listed journals, MDPI was the publisher with 74 journals with a continuous increase in published

articles over this time, followed by Springer (Biomed Central Ltd.) with 21 journals (Fig. 3c and Table S5)."

Comment #1.5: Lines 526-529 are unclear, specifically "the first was and the latter has been on Beall's list" - aren't these the same thing?

Answer #1.5: Thank you for the question. Frontiers was and still is on Beall's list. In contrast, MDPI was initially included in Beall's list, but it was later removed as MDPI effectively contested Beall's inclusion. To clarify this we reworded the sentence: „Other journals on Beall's list appear to not have been negatively affected by the increase of media coverage in 2018. Frontiers media, for example, was on Beall's list at the time of analysis and increasing their article count after 2018. The same is true for MDPI, which had been on Beall's list but was later removed from it."

Comment #1.6: The text in figures is very small - font size should be increased "

Answer #1.6: We thank the reviewer for this helpful suggestion and revised the figures 1 to 4, which are now labeled as Figures 1 to 8 to improve readability additionally to the increased font size in each figure.

Reviewer #3:

Comment #2.1: When tracing the changes I spotted only one fragment that needs minor correction. The citation in the last sentence of the paragraph on page 5 seems to be incorrect - Crawford uses both lists of journals but do not provide citation analysis.

"Although there has been some work done in analysing citations of articles from potential predatory journals, these analyses were limited to databases which had quality driven inclusion criteria.(12)"

Answer #2.1: We thank the reviewer for this important observation and included now the correct study: Björk, B.-C.; Kanto-Karvonen, S.; Harviainen, J.T. How Frequently

Are Articles in Predatory Open Access Journals Cited. Publications 2020, 8, 17.
https://doi.org/10.3390/publications8020017

Comment #2.2: Moreover, after revision the authors could also revise the beginning of the next paragraph for stylistic purposes.

"It remains, therefore, unknown how many journals on Beall’s list are included in quality-driven databases such as PubMed, how many articles are published by the journals on Beall’s list and how this number has changed over the past years, or how often articles published by journals on Beall’s list are referenced by other publications."

In the current version those two mentions of "quality driven" in almost opposite context one after another is confusing.

Answer #2.2: We agree with the reviewer and changed the phrase before the paragraph. „quality driven“ was replaced by „more rigorous“.
