# Supplementary material for: Predatory journals: Perception, impact and use of Beall’s list by the scientific community–A bibliometric big data study
Source: PLoS One. 2023 Jul 7;18(7):e0287547. doi: 10.1371/journal.pone.0287547 (PMC10328228; doi:10.1371/journal.pone.0287547)
Supplement: Attachment — Submitted filename: Comments on perception, impact and use of Bealls use .doc [file pone.0287547.s004.doc]

**Comment on the"Perception, impact and use of Beall’s list by Scientific Community**”

**General comments**

The title is good, and I got much information about Beall’s list. But I have some concerns especially regarding to methodology part. All the results except the exploratory analysis reported in the manuscript are related to binary logistic regression model, for instance, the odds ratio (OR) is the key element to interpret the results of binary logistic regression model. However, the authors used bibliometric approach to analyse the data. So how can you relate these two different concepts? Can we use bibliometric data analysis in place of binary logistic regression model?. No, as statistician, the two models are significantly different. Therefore, the authors should use the appropriate statistical model by inviting the experts such as statisticians. Secondly, all most all results of the paper are supported by exploratory (graphical) analysis, but the exploratory analysis are mainly used for descriptive not for inferential statistics. The conclusion and recommendations of any manuscript are mostly depending of the inferential statistics. So could you believe that your conclusion and recommendations came from the appropriate statistical analysis?. I pointed out this because making conclusion and recommendations based on descriptive statistics greatly affects the generalizability of your findings. Besides, the conclusion is not supported by the data in appropriate fashion. The manuscript also did not include the research ethics. Moreover, the statistical analysis is not appropriate and not described in detail.

**Specific comments**

|  | **Page number** | **Issues** | **Comments/suggestions** |
| --- | --- | --- | --- |
| 1 | Abstract  Page 2 | **Objectives:** We aim to investigate its impact on perception of listed journals as well as on the publication and citation ……… | How can you define or measure perception? It should be explicit. |
| 2 | Abstract  Page 2 | The authors took the data from ISSN databases, PubMed, PubMed Central (PMC), Crossref, Scopus and Web of Science. | What was the authors’ criterion to select those databases? You did not describe well in the methodology part. |
| 3 | Abstract  Page 2 | …..Beall’s list consisted of 1289 standalone journals, and 1162 publishers, which corresponds to a total of 21735 individual journals. Of these, 3206 (38.8%) were located in the United States, 2484 (30%) in India………. | What are sampling units in your manuscript? I understand that the sampling units are Journals. If so, your sample size is implicit. But, from this section/part, it seems that the authors used a sample size of 21735 journals. How did you get 38.8%?. Is it the ratio of 3206 to 21735 in percentage? Please cross check all the figures corresponding to their percentages. |
| 4 | Abstract  Page 2 | Journals on Beall’s list were more often cited when listed in web of Science (CI 95% 5.5 to 21.5; OR = 10.7) and PMC (CI 95% 6.3 to 14.1; OR = 9.4) | You used bibliometric analysis of data. Does odds ratio (OR) applicable for this approach?. OR is mainly used in logistic regression model to interpret the model parameters. In the situations of using OR the outcome/dependent variable should be binary or ordinal or nominal. In addition, OR needs explicit single reference (comparative group) to interpret the result. For example, in this section the authors said that, journals listed in Web of Science and PMC were more often cited than X (X should be single and clear). In addition, the interpretation of OR is also not clear. It should be rewrite as journals listed in Web of Science are 10.7 times more likely to cite than the reference group (X). Use the same fashion for all ORs through of the manuscript  Lastly, the OR and its confidence interval should be rewritten as  (95% CI: 5.5, 21.5, OR = 10.7).  And if you see the confidence interval carefully, it is very wide which intern results in biased results. What is the authors’ suggestion behind this wide confidence interval? Please keep all of these comments in the result section. |
| 5 | Abstract  page 2 | …..Journals are more likely to be selected for publication or citation when indexed by commonly used and renewed databases. | This result or interpretation is also related to Binary logistic regression model. However, the authors did not discuss about this model in the methodology part of the manuscript. |
| 6 | Results  Page 11-12 | The highest likelihood of cited by program participations was among journals listed in WoS (OR =10.7, 95% CI: 5.5 to 21.5), followed by PMC (OR = 9.4, 95% CI: 6.3 to 14.1) and ….. | The interpretation of OR is still not statistically correct. Pleases rewrite it and put the correct implication of OR.  Always the authors should underline that we cannot talk about odds ratio (OR) without logistic regression model. But I did not get any information and application of logistic regression model in your manuscript.  But I recommend that  The odds of being cited for journals listed in WoS were 10.7 times higher than that of the reference group. Similarly, the odds of being cited for journals listed in PMC were 9.5 times higher than that of the reference group.  I mentioned before, you did not define clearly the comparative (reference) group. |
| 7 | Results  Page 12 | Journals listed in PubMed, PMC or Scopus (n = 300) were cited 4455 times, on average, as compared to 231 journals for journals not listed in any of these databases (n=453). | Still the way of explanation or interpretation your result is incorrect. From this section, I understand that there is one outcome variable i.e. citation status (cited, not cited). Such types of outcome variables are treated with logistic regression especially by using binary logistic regression model. As a result all results should be revised. |
| 8 | Discussion | All discussion part | This section is relatively good. But it needs some modifications. |
| 9 | Conclusion | All conclusion part | Relatively good |
