# Supplementary material for: Predatory journals: Perception, impact and use of Beall’s list by the scientific community–A bibliometric big data study
Source: PLoS One. 2023 Jul 7;18(7):e0287547. doi: 10.1371/journal.pone.0287547 (PMC10328228; doi:10.1371/journal.pone.0287547)
Supplement: Attachment — Submitted filename: Rebuttal_letter_PONE-D-22-08372.R1_fsGR_MM_final.docx [file pone.0287547.s006.docx]

PONE-D-22-08372.R1

Dear Dr. Lopes,	February 2023

we thank the reviewers for their valuable comments and the Editorial Board for the consideration of our work for possible publication in PLOS One.

The following changes have been made according to the suggestions of Reviewer #3:

**Comment #1:** There is a need for clarification of what kind of Beall’s list’s „impact” authors aim to study. Eg. developing policies, hiring processes, national research evaluation systems, or discourse around OA are areas where the impact of Beall’s list can be measured. The authors seem to be focused only on very general publishing patterns of researchers - which is a valid aim of the study but should be stated more clearly. This publication could be helpful for authors in expanding the context of this impact:

Krawczyk, F., & Kulczycki, E. (2021). How is open access accused of being predatory? The impact of Beall’s lists of predatory journals on academic publishing. The Journal of Academic Librarianship, 47(2), 1–11. <https://doi.org/10.1016/j.acalib.2020.102271>

**Answer #1:** We gratefully thank the reviewer for this valuable suggestion and added the following specification to the introduction to make the aim of our study more clear in accordance to the suggested literature: “The aim of this paper is to characterize Beall’s list in total, to see where such journals/publisher came from and in which databases such journals/publisher were listed since such metrics are widely used by authors to decide where to publish. Secondly, we aimed to see how journals listed on Beall’s list performed – in terms of article count – in comparison to journals being listed in the DOAJ over the recent years. And thirdly, we investigate the number of citations that articles of journals being listed on Beall’s list received.”

**Comment #2:** Although they are right that: "Most studies performed on Beall’s list have limited their analyses either to only its listed journals, excluding listed publishers, or they investigated only a sample of the complete list.(5)", authors should better point out the originality of their research. The way they do it on Page 5 is not sufficient.

Writing about „most of the studies” is vague and they are not mentioning studies that are actually using both lists of journals and of publishers. Eg.:

Crawford, W. (2014). Journals, “Journals” and Wannabes: Investigating The List. Cites & Insights, 14(7).

Nelson, N., & Huffman, J. (2015). Predatory Journals in Library Databases: How Much Should We Worry? The Serials Librarian, 69(2), 169–192. https://doi.org/10.1080/0361526X.2015.1080782

**Answer #2.** We thank the reviewer for this supportive comment and revised the introduction section accordingly: “The majority of studies performed on Beall’s list have limited their analyses either to only its listed journals, excluding listed publishers, investigated only a sample of the complete list or used one of the earlier versions of the list.(8,12) Although there has been some work done in analysing citations of articles from potential predatory journals, these analyses were limited to databases which had quality driven inclusion criteria.(12)”

**Comment #3:** Moreover, the authors mention that it is unknown how many articles from predatory journals "are referenced by other publications” but they do not mention any existing study of citations to predatory journals and limitations of such studies which their study would overcome. See, for instance:

Frandsen, T. F. (2017). Are predatory journals undermining the credibility of science? A bibliometric analysis of citers. Scientometrics, 113(3), 1513–1528. https://doi.org/10.1007/s11192-017-2520-x

Kulczycki, E., Hołowiecki, M., Taşkın, Z., & Krawczyk, F. (2021). Citation patterns between impact-factor and questionable journals. Scientometrics. https://doi.org/10.1007/s11192-021-04121-8

The method used by the authors also seems to have some limitations from this perspective since they are able to calculate citations to only a small part of journals from the list (n = 812).

**Answer #3:** We agree with the reviewer that we haven’t clearly pointed out the potential advantages of our study and how it adds to previous analyses. To address this concern, we added wording on this to the limitation section of the discussion and highlighted the challenges of big data bibliometric studies: “Prior to our study, it was unclear how many articles are published by journals on Beall’s list and what has been the impact of Beall’s list on open-access journals in general, but due to the systematic bibliometric big-data approach, we received by far the biggest picture of this potential problem. We demonstrated that there was a constant increase in articles published by journals on Beall’s list and journals listed in the DOAJ until 2017 suggesting that authors who published in these journals may not have been aware of Beall’s list or ignored their listing.” Furthermore, we included the above mentioned studies into the discussion.

**Comment #4:** In my opinion, there is also a need to revise the first paragraph of the paper (page 1). The way in which Open Access is described is incorrect. Authors write: "In the open access (OA) model, authors pay an article-processing fee for the publishing services of a journal, and everyone can read the article." For a good description of the diversity of different modes of publishing in OA, I can recommend the works of Peter Suber, for instance: Suber, P. (2012). Open Access. MIT Press. http://mitpress.mit.edu/sites/default/files/titles/content/9780262517638_Open_Access_PDF_Version.pdf

**Answer #4:** We thank the reviewer for this well-taken point and agree that there are nowadays several different Open-access models (green OA, gold OA, hybrid OA,…) available but we tried to break it down and simplify the model for an easier understanding of the problem although – from a publishing point of view – it is not fully correct. We aimed to focus on the basic idea behind the open-access model in more detail in the introduction according to the review’s suggestion.

**Comment #5.** Moreover, describing journals financed mostly by subscriptions as „traditional” is problematic. When one looks at the history of academic publishing one can argue that a strong tradition of financing academic publishing mostly by philanthropy and scholarly societies exists. (Fyfe, A., Moxham, N., McDougall-Waters, J., & Røstvik, Camilla M. (2022). A History of Scientific Journals. UCL Press. <https://doi.org/10.14324/111.9781800082328>)

**Answer #5.** We thank the reviewer for raising this point, it is well taken. We completely reworded the first paragraph, bringing on board the suggestions of the reviewer and tried to summarize this complicated topic in a short overview.
“In the last decades, authors submitted their work for peer-review to be published in a paper-based journal. Costs were covered by a scientific society behind the journal, subscription fees to the journal, and/or industry sponsoring.(1) Importantly, access to articles is restricted to subscribers and those who pay per article. In the open-access (OA) model, articles are freely available to everyone who wants to read them.(2) However, the most common model of open-access is that authors pay a publisher an article-processing fee for the publishing services of a journal – including expenses for editing, distributing, hosting the article and peer-reviewing an article – and therefore for the option to make their article globally available at no cost for the reader. The transition of the open-access model in science has been performed in many different ways with many different OA models including green OA, Diamond OA, Gold OA, Bronze OA and many more.(3)“

**Comment #6.** There is a need for more clarity in the section "Beall’s list did not significantly impact the Open Access movement”. The one issue is that it is not exactly clear when authors describe in this section journals from Beall’s list and when open access journals from DOAJ. The second issue is connected to a very specific understanding of impact - the authors are studying only very general publishing patterns of academics, but writing about „impacting Open access movement” may suggest that studied qualitatively how OA is perceived (eg. Scholars can publish in journals they dislike to fulfill the number of publications university require from them).

**Answer #6.** We thank the reviewer for raising this very important point. The aim of this section was to see if the discussion around Beall's list and the open-access movement had a general impact on the open-access model. A negative association with the open-access movement should also have led to a reduced article count and therefore harmed the idea of the open-access movement. We tried to clarify this section by providing a more clear introduction section within the paragraph: “To assess the impact of Beall’s list on Open-Access publishing as well as on journals’ publishing output of journals on Beall’s list, we compared the article count of journals on Beall’s list in comparison to journals on the DOAJ.”

We further added the following sentence to the discussion section to further highlight the problem of predatory journals and our study’s limitations.

“In addition, there may be scientists who actively use predatory journals to publish their scientific work as fast as possible – independently of the scientific quality of the work – to fulfill the publication requirements of their universities.(24) In this study we only assessed the numbers of publications rather than the motivation of publishing in predatory journals and this can be – as mentioned above – due to multiple reasons.“

**Comment #7.** I believe better arguments and a reference are needed to support the statement that "Web of Science has the most rigorous criteria for inclusion in their database” on page 21. It is not entirely clear what „rigorous” mean in this context - eg. DOAJ is listing only open access journals and WoS is open also for subscription-based journals.

**Answer #7.** Thank you for this helpful feedback. We refined our statement in the manuscript and included more detailed information on inclusion criteria in Web of Science and why they were more “rigorous”. The paragraph was reworded and expanded to:

“Another important criterion of the scientific quality of a journal is its listing in databases. Several studies could demonstrate that potential predatory journals are indexed in databases like pubmed, scopus and web of science.(18) Journals can apply for inclusion by a specific database and, since different databases have different inclusion criteria, it makes a significant difference in which database a journal is listed. Since Web of Science only wants to cover the ‘core journals’ in a specific scientific category, each applicant is assessed by an in-house editorial team, the use of publicly available criteria, and with the help of citation analysis.(19) A similar rigorous inclusion process is used by Scopus, which is hosted by Elsevier.(19) This makes it harder for journals/publisher to be included in such databases, and, therefore, it was no surprise that the number of Beall’s journals listed there was the lowest.“

**Comment #8.** The article would have benefited if its results would be put in the wider context of the other research and their limitations in the discussion section. Eg. how authors’ interesting analysis of titles journals and locations of journals can be compared to the "Comparison of journal website contact location claims and actual locations based on the IP/WHOIS” conducted in Demir, S. B. (2018). Predatory journals: Who publishes in them and why? Journal of Informetrics, 12(4), 1296–1311. https://doi.org/10.1016/j.joi.2018.10.008

**Answer #8.** We are grateful for these very valuable comments and have incorporated the suggested studies in the discussion section and also elaborated on limitations and differences in the context of our study. We have revised several paragraphs in our manuscript to provide more detailed information on the broader context of our study compared to other research. We truly believe that the valuable comments and suggestions given by the reviewer helped to critically improve our manuscript and to facilitate the digestion of the topic by the reader.

“In our study, the majority of journals were located in the United States followed by India, which is in contrast to a study published by Demir, who could demonstrate that the majority of journals on Beall’s list came from India.(17) This divergence can be explained by the fact that he only used a small part of Beall’s list and made a IP/WHOIS (which only gives the location of the hosting institution/VPN rather than the organization behind it), whereas we used the entry of the ISSN database to identify the country of origin.”

“Since Web of Science only wants to cover the ‘core journals’ in a specific scientific category, each applicant is assessed by an in-house editorial team, the use of publicly available criteria, and with the help of citation analysis.(19) A similar rigorous inclusions process is used by Scopus, which is hosted by Elsevier.(19) This makes it harder for journals/publisher to be included in such databases, and, therefore, it was no surprise that the number of Beall’s journals listed there was the lowest”

“Another important question was if articles from journals on Beall’s list actually get any citations. Therefore, we used crossref’s cited-by-database to estimate the number of citations. The advantage of our study in comparison to previous studies was that there was a low entry barrier for journals/publisher to provide such information. Although it provides only a small slice of the problem, it is a larger one than in previous studies, but results are still highly dependent on the information provided.(29) Importantly we could demonstrate that journals on Beall’s list get indeed citations, and this was in high dependency of the databases such journals were listed in. Therefore, articles from journals listed in Web of Science were more often cited. In return - as shown by another group – even journals listed in Web of Science cite such articles.(30)”
